# Dispersal capacities of pollen, seeds and spores: insights from comparative analyses of spatial genetic structures in bryophytes and spermatophytes

**DOI:** 10.3389/fpls.2023.1289240

**Published:** 2023-10-30

**Authors:** T. Fichant, A. Ledent, F. Collart, A. Vanderpoorten

**Affiliations:** ^1^ Institute of Botany, University of Liège, Liège, Belgium; ^2^ Department of Ecology and Evolution, University of Lausanne, Lausanne, Switzerland

**Keywords:** spatial genetic structure, geographic barriers, phylogeographical signal, GST, NST, Mediterranean, nDNA, cpDNA

## Abstract

**Introduction:**

The dramatic fluctuations of climate conditions since the late Tertiary era have resulted in major species range shifts. These movements were conditioned by geographic barriers and species dispersal capacities. In land plants, gene flow occurs through the movement of male gametes (sperm cells, pollen grains), which carry nDNA, and diaspores (spores, seeds), which carry both cpDNA and nDNA, making them an ideal model to compare the imprints of past climate change on the spatial genetic structures of different genomic compartments. Based on a meta-analysis of cpDNA and nDNA sequence data in western Europe, we test the hypotheses that nDNA genetic structures are similar in bryophytes and spermatophytes due to the similar size of spores and pollen grains, whereas genetic structures derived from the analysis of cpDNA are significantly stronger in spermatophytes than in bryophytes due to the substantially larger size of seeds as compared to spores.

**Methods:**

Sequence data at 1-4 loci were retrieved for 11 bryophyte and 17 spermatophyte species across their entire European range. Genetic structures between and within southern and northern populations were analyzed through F and N statistics and Mantel tests.

**Results and discussion:**

Gst and Nst between southern and northern Europe derived from cpDNA were significantly higher, and the proportion of significant tests was higher in spermatophytes than in bryophytes. This suggests that in the latter, migrations across mountain ranges were sufficient to maintain a homogenous allelic structure across Europe, evidencing the minor role played by mountain ranges in bryophyte migrations. With nDNA, patterns of genetic structure did not significantly differ between bryophytes and spermatophytes, in line with the hypothesis that spores and pollen grains exhibit similar dispersal capacities due to their size similarity. Stronger levels of genetic differentiation between southern and northern Europe, and within southern Europe, in spermatophytes than in bryophytes, caused by higher long-distance dispersal capacities of spores as compared to seeds, may account for the strikingly higher levels of endemism in spermatophytes than in bryophytes in the Mediterranean biodiversity hotspot.

## Introduction

In land plants, assessing dispersal is a challenging issue that can be approached either directly or indirectly (Bullock et al., 2006). Direct techniques involve actual measurements of diaspore dispersal and subsequent modelling of the probability of dispersal as a function of the distance from the source (the dispersal kernel) (see Clobert et al., 2012 for review). Indirect techniques are based on inferences from spatial genetic structures (e.g. Vekemans and Hardy, 2004), which are shaped by the dispersal of male gametes (sperm cells, pollen grains) and diaspores (spores, asexual diaspores, seeds). In spermatophytes and bryophytes, chloroplast DNA (cpDNA) is mostly maternally inherited and therefore dispersed only via seeds, spores and asexual diaspores. Nuclear DNA (nDNA), in contrast, has a biparental inheritance. It is thus dispersed by both seeds and pollen grains in spermatophytes, and spores, asexual diaspores and sperm cells in bryophytes. The contribution of gametes and diaspores to gene flow can hence be disentangled by using markers of different inheritance.

In spermatophytes, variations in dispersal capacities are intimately linked to morphological traits of diaspores related to particular dispersal vectors, referred to as dispersal syndromes (Arjona et al., 2018). In anemochorous species in particular, the range, at which seeds disperse, is correlated with their ornamentation patterns but also with their size, smaller particles dispersing further than larger ones due to their lower settling velocity, and thus, longer expected airborne time (Bullock et al., 2006). Seed size ranges between 0.05 mm (Arditti and Ghani, 2000) to tens of centimetres (Morgan et al., 2017), largely exceeding the size of pollen grains, which range between 10 and about 100 µm in diameter (Hao et al., 2020). Seeds and pollen grains have different potential for dispersal (Santos et al., 2018), so that genetic variation in cpDNA is typically much more spatially structured than in nDNA (Petit et al., 2005). In bryophytes, sperm cells must swim to the archegonia through a continuous film of water, so that fertilization distances are typically very short, <30 cm in mosses up to about 20 m in complex thalloid liverworts equipped with specialized antheridial ‘splash cups’ (Pressel and Duckett, 2019). Therefore, one can hypothesize that, in bryophytes, gene flow mostly occurs via spores, which mostly measure about 10-20 µm in diameter, whose release is facilitated by the hygroscopic movements of specific structures, namely the peristome in mosses and elaters in liverworts, and which are mostly wind-dispersed. The long-distance dispersal capacities of bryophyte spores have had a substantial impact on their biogeographic history and extant distribution patterns, as evidenced by their much lower rates of endemism, larger, trans-oceanic distribution ranges (Patiño and Vanderpoorten, 2018), and lower species turn-over among communities (Patiño et al., 2014b; Mouton et al., 2023) than in spermatophytes. To our knowledge, however, no study has so far attempted at determining whether the putatively higher dispersal capacities of bryophytes as compared to spermatophytes has impacted their spatial genetic structure.

The massive species distribution range shifts that took place due to fluctuations of climate conditions since the late Tertiary era (Hewitt, 1996; Comes and Kadereit, 1998; Hewitt, 1999; Hewitt, 2000; Hewitt, 2003; Petit et al., 2003; Hewitt, 2004; Lumibao et al., 2017) offer an appealing framework to assess how plant species migrated as a response to climate change. In Europe, palaeontological and phylogeographic evidence suggest that species either persisted in scattered southern refugia, wherein populations evolved in isolation, generating high genetic differentiation among them (Hewitt, 1999; Hewitt, 2000; Hewitt, 2004; Médail and Diadema, 2009), or in micro-refugia located in the steppe zone South of the ice sheet (Bhagwat and Willis, 2008). During warmer periods, populations expanded northward from southern refugia or northern micro-refugia, generating admixed populations with high genetic diversity at mid-latitudes, and genetically depauperate populations at high latitudes resulting from long-distance dispersal events and associated founder effects (Hewitt, 1999; Petit et al., 2003).

These movements have been hampered by East-West oriented mountain ranges, limiting genetic exchange between populations located South and North of the barrier and resulting in differences in allele frequencies among populations (Petit et al., 2003). When dispersal was limited to such an extent, that dispersal rates have been lower than migration rates, sister alleles tended to be distributed closer to each other than distantly related alleles, generating phylogeographic signal (Pons and Petit, 1996) potentially leading, ultimately, to allopatric speciation.

Here, we performed a meta-analysis of the spatial genetic structures of bryophytes and spermatophytes across Europe to address the following questions and test the following hypotheses:

- To what extent have mountain ranges hampered post-glacial colonization of the continent in bryophytes and spermatophytes? We hypothesize that genetic divergence between southern and northern populations is higher in spermatophytes than in bryophytes (H1). We further hypothesize that these differences are reflected by the presence of a phylogeographical signal in spermatophytes, wherein sister alleles are more likely to occur in sympatry, whereas in bryophytes, dispersal rates would exceed mutation rates, breaking the phylogenetic structure of genetic variation across the continent (H2).
- To what extent have differences in dispersal capacities between bryophytes and spermatophytes shaped differences in their spatial genetic structure on both sides of the mountain barriers? We hypothesize that the slope of the regression between genetic similarity and geographic distance is significantly higher in spermatophytes than in bryophytes and that distance is a better predictor of genetic similarity in spermatophytes than in bryophytes due to faster recolonization by random long-distance dispersal events since the last glacial maximum in the latter (H3).
- These differences vary depending on the analysis of cpDNA vs nDNA variation. We hypothesize that H1, H2 and H3 apply using cpDNA markers, whereas the spatial genetic structure derived from the analysis of nDNA markers are similar between bryophytes and spermatophytes due to similar sizes of spores and pollen grains (H4).

## Materials and methods

**Figure 1:**
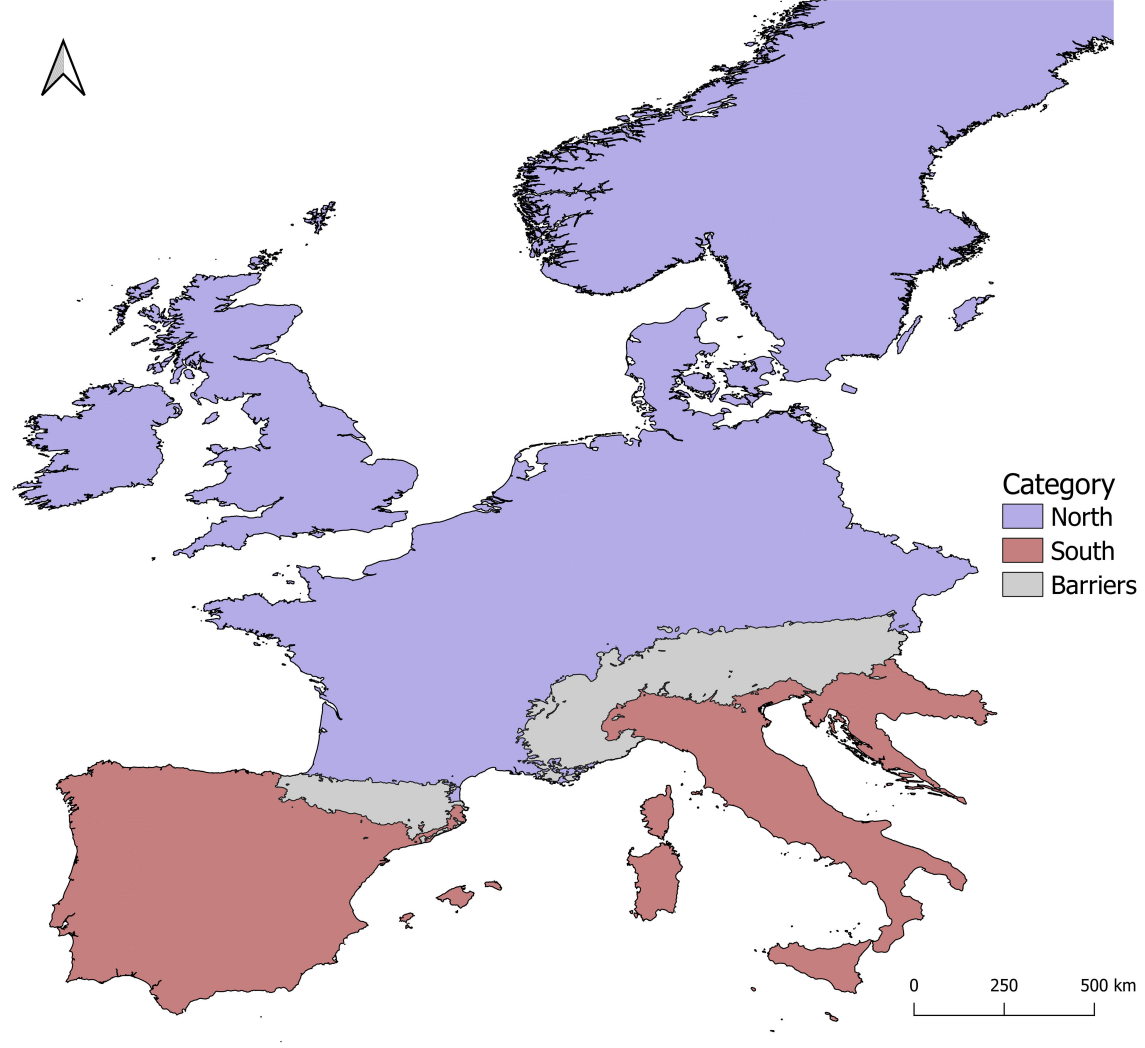
Study area (western Europe) and its division into northern and southern regions on both sides on the mountain ranges of the Alps and the Pyrenees.

This study focused on Western Europe, as circumscribed eastwards by the easternmost margin of the Alps, i.e., ~15.00 decimal degrees of longitude. The area was divided into South and North, delimited by the Pyrenees and the Alps (**Figure 1**). A search of plant phylogeographic and population genetic structure in Europe was performed using Scopus, employing phylogeography or phylogeographic, genetic structure or geographic structure, Europe or European, spermatophyte or bryophyte, as keywords. We focused on studies employing DNA sequence data, which allow for an assessment of the genetic divergence among alleles, and hence, to test for phylogeographical signal (H2). From an initial number of 188 datasets, 160 were removed due to limited sampling (here set at a minimum of 15 specimens at each locus), leaving a total of 17 and 11 spermatophyte and bryophyte datasets, respectively (**Table S1**).

Individual sequences were retrieved from GenBank and aligned with the ‘muscle’ algorithm (Edgar, 2004) as implemented in Seaview v5.0.4 (Gouy et al., 2010). In all analyses, cpDNA data were concatenated and considered as one locus, whereas each nuclear locus was considered individually. Regions of ambiguous alignment were removed with Gblocks (v0.91b) (Talavera and Castresana, 2007) as follows: the minimum length of a block after gap cleaning was set to 5. Positions with a gap in less than 50% of the sequences were selected in the final alignment if they were within an appropriate block. All segments including more than 8 contiguous nonconserved positions were rejected. Finally, the minimum number of sequences for a flank position was 85%. Pairwise genetic distances between haplotypes were computed using Kimura’s 2-parameters distance substitution model in the R environment using the package ape 5.6-2 (Paradis and Schliep, 2019). Indels were treated as missing data.

To test the impact of geographic barriers on genetic structure, Gst and Nst were computed between the northern and southern areas. A standard deviation was computed for multilocus datasets by a Jackknife test, wherein Gst and Nst were recalculated after successively pruning one locus from the data at a time as implemented by Spagedi (Hardy and Vekemans, 2002). Presence of a geographic partitioning of allele frequencies between the North and the South was tested by 1000 random permutations of individuals among regions. To test the hypothesis that Gst is, on average, higher in spermatophytes than in bryophytes (H1), t-tests assuming unequal variances were implemented.

We sought for phylogeographic signal in the data by testing the hypothesis that Nst > Gst (H2). Nst is a measure of genetic differentiation among populations analogous to Gst but taking into account the phylogenetic relationships among alleles (Pons and Petit, 1996). An interesting property is that Nst > Gst when phylogeographical signal exists; that is, when distinct alleles sampled within populations are phylogenetically closer, on average, than alleles sampled from different populations (Pons and Petit, 1996). The hypothesis that Nst > Gst was tested by computing the distribution of the null hypothesis (no phylogenetic structure of allelic differentiation among regions) by conducting 1000 permutations of the matrix of genetic distance among alleles.

To test our third hypothesis (H3) that the slope of the regression between genetic similarity and geographic distance is significantly higher in spermatophytes than in bryophytes and that distance is a better predictor of genetic similarity in spermatophytes than in bryophytes, we regressed pairwise kinship coefficients Fij (Ritland, 1996) between individuals and pairwise geographic distances. In these conditions, populations were not continuous, and presence of a significant slope was interpreted in terms of a significant geographic structure of genetic variation resulting from either presence of a geographic barrier or actual isolation-by-distance. We determined whether log-transforming the distance significantly improved the r^2^ of the Mantel test by comparing the r^2^ obtained using linear and log-transformed distances using a paired t-test. Significance of the slope was tested by 1000 random permutations of individuals among localities (Mantel test). We further estimated the standard deviation of the slope by implementing a Jackknife test as described above.

To test the hypothesis that the spatial genetic structures of bryophytes and spermatophytes differ with cpDNA due to the larger size of seeds as compared to spores, but not with nDNA due to the similar size of spores and pollen grains (H4), all analyses were performed for cpDNA, nDNA, and both markers combined, except the Mantel tests between geographic distance and Fij derived from nDNA due to missing geographic coordinates for individuals in two spermatophyte species (*Arabidopsis thaliana* and *Beta vulgaris*), leaving only four spermatophyte species with nDNA data available for this comparison.

## Results

Gst between populations of northern and southern Europe ranged in bryophytes between 0 and 0.17 (excluding an outlier value of 0.77 in *Pulvigera lyellii* (Hook. & Taylor) Plášek, Sawicki & Ochyra due to the very clear segregation of alleles, see **Figure S7**), 0.02 and 0.29, and 0.05 and 0.25 with cpDNA markers, nDNA markers, and both combined (**Table 1**), respectively. In spermatophytes, the range of Gst was 0-0.51, 0.02-0.14 and 0.02-0.29 with cpDNA markers, nDNA markers, and both combined, respectively. (**Table 1**). On average, Gst was significantly higher in spermatophytes than in bryophytes with cpDNA (Gst = 0.06 ± 0.05 and 0.16 ± 0.17 in bryophytes and in spermatophytes, respectively, p<0.05), but not with nDNA (Gst = 0.09 ± 0.11 and 0.09 ± 0.05 in bryophytes and in spermatophytes, respectively, p=0.46) and both markers combined (Gst = 0.12 ± 0.10 and 0.11 ± 0.11 in bryophytes and in spermatophytes, respectively, p=0.43). Significant Gst values were found in two out of the 11 bryophyte species considered for cpDNA markers, nDNA markers, and both combined (**Table 1**). A higher proportion of significant test was obtained in spermatophytes, wherein 8, 2 and 2 Gst values derived from the analysis of cpDNA markers, nDNA markers, and both combined, respectively, were found (**Table 1**).

**Table 1:** Summary statistics of the genetic structure (Gst, Nst) of 11 bryophyte and 17 spermatophyte species between northern and southern Europe based on cpDNA, nDNA sequence data and both combined.

Species (number of individuals)	Partition (number of loci)	Gst	P-Gst	Gst-Jack	Nst	P-Nst	Nst-Jack	P(Nst>Gst)
Bryophytes								
*Antitrichia curtipendula* (37)	Both (2)	0.05	0.28	0.05 ± 0.04	0.06	0.18	0.09 ± 0.06	0.21
	nDNA (1)	0.03	0.34	NA	0.07	0.27	NA	0.48
	cpDNA (1)	0.07	0.63	NA	0.00	0.01	NA	0.33
*Amphidium mougeotii* (52)	nDNA (2)	0.02	0.49	0.02 ± 0.06	0.00	0.69	0.00 ± 0.00	0.29
*Calypogeia fissa* (26)	cpDNA (1)	0.04	0.52	NA	0.01	0.76	NA	0.83
*Homalothecium sericeum* (46)	cpDNA (1)	0.11	0.03	NA	0.08	0.06	NA	0.86
*Metzgeria furcata* (59)	cpDNA (1)	0.00	0.82	NA	0.00	0.60	NA	0.63
*Lewinskya affine* (57)	Both (4)	0.05	0.17	0.05 ± 0.10	0.03	0.28	0.03 ± 0.05	0.44
	nDNA (3)	0.06	0.15	0.06 ± 0.11	0.03	0.25	0.03 ± 0.05	0.45
	cpDNA (1)	0.00	0.88	NA	0.00	0.88	NA	0.20
*Plagiomnium undulatum* (57)	Both (3)	0.21	0.00	0.20 ± 0.17	0.34	0.00	0.35 ± 0.07	0.03
	nDNA (2)	0.30	0.00	0.30 ± 0.05	0.37	0.00	0.37 ± 0.03	0.03
	cpDNA (1)	0.08	0.37	NA	0.13	0.29	NA	0.48
*Plagiothecium undulatum* (42)	nDNA (2)	0.02	0.60	0.02 ± 0.14	0.00	1.00	0.03 ± 0.17	0.17
*Pulvigera lyellii* (81)	Both (4)	0.26	0.00	0.28 ± 0.46	0.28	0.00	0.31 ± 0.46	0.52
	nDNA (3)	0.02	0.44	0.02 ± 0.04	0.04	0.15	0.05 ± 0.09	0.35
	cpDNA (1)	0.77	0.00	NA	0.77	0.00	NA	0.80
*Scorpiurium circinatum* (18)	Both (2)	0.15	0.09	0.15 ± 0.16	0.04	0.50	0.10 ± 0.13	0.06
	nDNA (1)	0.22	0.05	NA	0.05	0.50	NA	0.07
	cpDNA (1)	0.06	0.48	NA	0.00	0.86	NA	0.14
*Sphagnum fimbriatum* (34)	cpDNA (1)	0.17	0.52	NA	0.20	0.06	NA	0.33
Spermatophytes								
*Arabis alpina* (38)	Both (2)	0.00	0.71	0.00 ± 0.04	0.07	0.17	0.08 ± 0.04	0.05
	nDNA (1)	NA	NA	NA	NA	NA	NA	NA
	cpDNA (1)	0.00	0.85	NA	0.05	0.44	NA	0.13
*Alnus glutinosa* (198)	cpDNA (1)	0.46	0.00	NA	0.42	0.00	NA	0.77
*Aegilops geniculata* (63)	cpDNA (1)	0.08	0.09	NA	0.22	0.01	NA	0.35
*Arabidopsis thaliana* (130)	Both (3)	0.08	0.00	0.08 ± 0.07	0.07	0.00	0.06 ± 0.09	0.76
	nDNA (2)	0.06	0.00	0.06 ± 0.11	0.10	0.00	0.08 ± 0.17	0.14
	cpDNA (1)	0.18	0.00	NA	0.02	0.18	NA	0.03
*Beta vulgaris maritima* (19)	Both (4)	0.06	0.09	0.06 ± 0.12	0.00	0.77	0.00 ± 0.08	1.00
	nDNA (3)	0.10	0.05	0.09 ± 0.16	0.01	0.76	0.00 ± 0.15	1.00
	cpDNA (1)	0.00	0.73	NA	0.00	0.69	NA	0.53
*Carex extensa* (52)	cpDNA (1)	0.25	0.01	NA	0.25	0.01	NA	1.00
*Ceratonia siliqua* (124)	cpDNA (1)	0.51	0.00	NA	0.55	0.00	NA	0.16
*Calluna vulgaris* (132)	cpDNA (1)	0.09	0.00	NA	0.36	0.00	NA	0.00
*Hedera helix* (27)	cpDNA (1)	0.22	0.01	NA	0.35	0.02	NA	0.40
*Hordeum marinum* (94)	cpDNA (1)	0.01	0.36	NA	0.02	0.37	NA	0.73
*Helianthemum nummularium* (26)	cpDNA (1)	0.01	0.67	NA	0.01	0.57	NA	0.81
*Lavatera maritima* (62)	cpDNA (1)	0.06	0.11	NA	0.10	0.04	NA	0.16
*Myrtus communis* (158)	Both (3)	0.02	0.07	0.03 ± 0.05	0.04	0.23	0.05 ± 0.07	0.82
	nDNA (2)	0.02	0.07	0.03 ± 0.07	0.04	0.19	0.05 ± 0.07	0.83
	cpDNA (1)	0.03	0.15	NA	0.06	0.04	NA	0.54
*Microthlaspi perfoliatum* (153)	Both (2)	0.30	0.00	0.33 ± 0.22	0.21	0.00	0.21 ± 0.02	1.00
	nDNA (1)	0.15	0.00	NA	0.21	0.00	NA	0.22
	cpDNA (1)	0.38	0.00	NA	0.19	0.01	NA	0.07
*Primula vulgaris* (21)	Both (2)	0.09	0.11	0.08 ± 0.06	0.00	0.89	0.00 ± 0.03	0.07
	nDNA (1)	0.12	0.21	NA	0.00	0.93	NA	0.05
	cpDNA (1)	0.06	0.31	NA	0.02	0.75	NA	0.79
*Sedum album* (24)	cpDNA (1)	0.02	0.38	NA	0.06	0.16	NA	0.40
*Silene nutans* (258)	cpDNA (1)	0.30	0.00	NA	0.28	0.03	NA	0.95

P-Gst and P-Nst are the p-values of the null hypothesis that Gst and Nst=0, respectively, tested by random permutations of individuals among regions. Gst-Jack and Nst-Jack are the mean ± 1.96 SD of these statistics after Jackknife across loci for multilocus data (NA otherwise). P(Nst>Gst) is the p-value of the null hypothesis that Nst>Gst, tested by random permutations of the matrix of genetic distance among alleles.

Nst in bryophytes ranged between 0 and 0.20 (excluding an outlier value of 0.77 in *Pulvigera lyellii*), 0 and 0.37, and 0 and 0.34 with cpDNA markers, nDNA markers, and both combined, respectively (**Figure 1**). In spermatophytes, the range of Nst was 0-0.55, 0-0.21 and 0-0.21 with cpDNA markers, nDNA markers, and both combined (**Figure 1**). On average, Nst between the North and the South were significantly higher in spermatophytes than in bryophytes with cpDNA (Nst=0.05 ± 0.08 and 0.17 ± 0.17 in bryophytes and spermatophytes, respectively, p<0.05), but not with nDNA and both markers combined (**Table 1**). Significant Nst values were found in 2 cpDNA, 1 nDNA and 2 combined bryophyte datasets. Again, the proportion of significant Nst values was higher in spermatophytes, with 10, 1 and 1 significant tests for cpDNA, nDNA, and both combined, respectively.

A significant phylogeographical signal (Nst>Gst) was found in 1 and 2 of the bryophyte and spermatophytes species investigated, respectively (**Table 1**).

**Figure 2:**
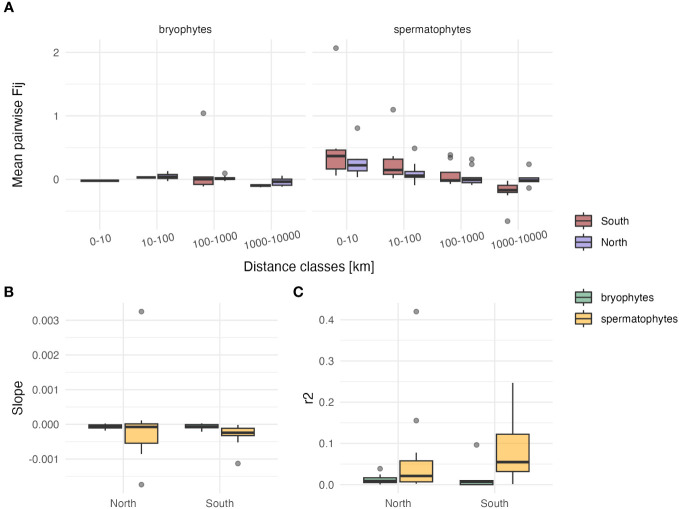
Comparison of isolation by distance patterns among individuals based on cpDNA sequence data between bryophytes and spermatophytes in southern (n=5 and n=12 in bryophytes and spermatophytes, respectively) and northern Europe (n=8 and n=12 in bryophytes and spermatophytes, respectively). **(A)** Box-plots (showing the first and third quartiles (upper and lower bounds), second quartile (center), 1.5* interquartile range (whiskers) and minima–maxima beyond the whiskers) of the mean kinship coefficient (Fij) between pairs of individuals as a function of geographic distance. **(B)** Box-plots of the slope of the regression between Fij and geographic distances. **(C)** Box-plots of the r^2^ of this relationship.

Log-transforming geographic distances in Mantel tests did not improve the r^2^ as compared to linear distances (for bryophytes: r^2^ linear=0.02 ± 0.04, r^2^ log=0.06 ± 0.13, p=0.28 in the South; r^2^ linear=0.01 ± 0.01, r^2^ log=0.01 ± 0.01, p=0.29 in the North; for spermatophytes: r^2^ linear=0.09 ± 0.09, r^2^ log=0.11 ± 0.09, p=0.28 in the South; r^2^ linear=0.07 ± 0.12, r^2^ log=0.06 ± 0.12, p=0.46 in the North). The average (± SD) slope of the regression between Fij derived from cpDNA markers and geographic distance was about four times steeper in spermatophytes than in bryophytes in the South (b=-7.16 10^-5^ ± 9.35 10^-5^ in bryophytes, b=-2.94 10^-4^ ± 3.0 10^-4^ in spermatophytes, p<0.05), but did not significantly differ in the North (b=-6.68 10^-5^ ± 6.74 10^-5^ in bryophytes, b=-7.13 10^-5^ ± 1.2 10^-4^ in spermatophytes, p=0.49) (**Figure 2**; **Table S2**). The r^2^ of these regressions was higher in spermatophytes than in bryophytes, but the difference was significant only in the South (South: r^2 ^= 0.09 ± 0.09 and r^2 ^= 0.02 ± 0.04 in spermatophytes and in bryophytes, respectively, p<0.05; North: r^2 ^= 0.07 ± 0.12 and r^2 ^= 0.01 ± 0.01 in spermatophytes and in bryophytes, respectively, p=0.07) (**Table S2**).

## Discussion

While differences in long-distance dispersal capacities between bryophytes and spermatophytes have long been discussed in terms of community assembly rules (for instance, in terms of ecological vs historical factors shaping variation of species composition (Patiño et al., 2013) or of variation of the shape of the species-area relationship due to differences in turnover rates among communities (Patiño et al., 2014b), the present results provide a first explicit basis of comparison to determine how such differences have shaped differences in their extant patterns of genetic structure. In agreement with our first hypothesis (H1), Gst and Nst between populations located in southern and northern Europe derived from cpDNA were significantly higher, and the proportion of significant tests was higher in spermatophytes than in bryophytes. In spermatophytes, the stronger North/South geographic structure is consistent with the idea that mountain ranges hampered post-glacial seed dispersal (Petit et al., 2003). In bryophytes, the fact, that a significant difference in allele frequencies between the North and the South (Gst>0) was found in only two out of the 11 investigated species, indicates that migrations across mountain ranges separating the North and the South were sufficient to maintain a homogenous allelic structure across Europe, evidencing the minor role played by mountain ranges in bryophyte migrations. These results are reminiscent of the minor role played by geographic barriers in mosses, wherein populations disjunct on both sides of an oceanic barrier exhibited the same levels of genetic differentiation as populations separated by similar distances but located on the same continent (Grundmann et al., 2007; Hutsemékers et al., 2011; Kyrkjeeide et al., 2016).

Nevertheless, and in contrast with our second hypothesis (H2), evidence for phylogeographical signal in the data was virtually absent for both bryophytes and spermatophytes. While in bryophytes, absence of a phylogeographical signal in the data was expected due to the large contribution of migrants of extra-European origin (Ledent et al., 2019), thereby breaking any phylogenetic structure of the spatial variation of alleles, we initially expected to observe sympatric diversification on both sides of the mountain barriers in spermatophytes. While such a signal was reported in European spermatophytes in specific areas, where they evolve in isolation (e.g., *Epipactis helleborine* (L.) Crantz in the Italian peninsula (Tranchida-Lombardo et al., 2011), *Salvia officinalis* L. in the Balkans (Stojanović et al., 2015), the absence of such a signal in the present results illustrates the fact that recolonization of northern areas did not take place from the diversification of a common ancestor, but instead, from several recolonization events from different refugia (Petit et al., 2003).

There was, however, a notable exception in the patterns described above, caused by the strikingly high value of the Gst of 0.77 derived from the analysis of cpDNA variation in the moss *Pulvigera lyellii* due to the almost complete allele segregation between northern and southern Europe (**Figure S7**), while Gst ranged between 0.00 and 0.05 across the tree nDNA loci sequenced for that species. By comparison, Korpelainen et al. (2005) reported an average Gst derived from the analysis of sequence data of 0.12 ± 0.11 in a review of the levels of genetic differentiation in bryophytes. While a higher structure in cpDNA is expected in spermatophytes due to different sizes, and hence, dispersal capacities between seeds and pollen grains, no such difference is expected in bryophytes, wherein sperm cells have an extremely limited dispersal range, with a maximum fertilization distance of <30cm in mosses up to about 20m in complex thalloid liverworts equipped with specialized antheridial ‘splash cups’ (Pressel and Duckett, 2019), thereby hardly contributing to gene flow at large spatial scales. We suggest that two processes may account for the striking level of cpDNA variation in *P. lyellii*. First, genetic drift, which is stronger in the chloroplast than in the nuclear genome (Ness et al., 2016), may not be balanced by migration. A dioicous moss species, *P. lyellii* indeed reproduces almost exclusively vegetatively in Europe (Zuijlen et al., 2023) via specialized asexual propagules, which have long been thought, due to their larger size and sensitivity to harsh conditions, to disperse much less effectively than spores (see Patiño and Vanderpoorten, 2018 for review). Such a hypothesis is, however, weakened by two lines of evidence. First, the idea, that vegetative diaspores are involved in short-distance dispersal, whereas spores are involved in long-distance dispersal, has been challenged (Pohjamo et al., 2006; Laenen et al., 2016). Second, other dioicous species included in the present study and also largely failing to reproduce sexually, such as *Plagiomnium undulatum* (Hedw.) T.J. Kop., did not display the same levels of spatial structure in cpDNA. Alternatively, the high cpDNA structure in *P. lyellii* could be explained in terms of selection. Recent evidence suggests that thermal adaptation may occur even in species with high gene flow (Evans et al., 2023), raising the hypothesis that, as opposed to previous assumptions (see Patiño et al., 2014a for review), bryophyte species may adapt locally, which would have considerable implications regarding predictions of their sensitivity to climate change that have assumed climate niche conservatism (Zanatta et al., 2020).

A stronger geographic structuring of cpDNA genetic variation in spermatophytes than in bryophytes was also found within southern Europe. In fact, in agreement with our third hypothesis (H3), the slope of the regression between kinship coefficients derived from cpDNA variation and geographic distance in southern Europe was about four times higher in spermatophytes than in bryophytes, and geographic distance was a significantly better predictor of this relationship, as evidenced by significantly higher r^2^ in spermatophytes than in bryophytes. In northern Europe in contrast, the differences of genetic structure between bryophytes and spermatophytes, as expressed by the slope and r^2^ of the regression between genetic similarity and geographic distance, was not significant. In spermatophytes, the strong levels of genetic divergence among populations point to an evolution in isolation in the different peninsulas over long periods of time (Petit et al., 2003). In bryophytes, genetic differentiation among populations within the Mediterranean is less pronounced due to the comparatively higher dispersal capacities of the group, especially for migrations above the sea (Hutsemékers et al., 2011; Kyrkjeeide et al., 2016), as further suggested by the comparatively low Fst values reported (for example, Fst=0.13 between E and W Mediterranean in *Homalothecium meridionale* (M. Fleisch. & Warnst.) Hedenäs (Désamoré et al., 2012), Fst=0.18 and 0.10 across the Mediterraneo-Atlantic region in *Rhynchostegium riparioides* (Hedw.) Cardot and *Radula lindenbergiana* Gottsche ex C. Hartm., respectively (Hutsemékers et al., 2011; Laenen et al., 2011)). In the North, the lack of significance of the genetic structure between bryophytes and spermatophytes suggests that a rapid post-glacial recolonization took place in both groups in the absence of major geographic barriers. Altogether, stronger levels of genetic differentiation between southern and northern Europe, and within southern Europe, in spermatophytes than in bryophytes, may account for the strikingly higher levels of endemism in the former than in the latter. This is especially true in the Mediterranean, the world’s second largest biodiversity hotspot, wherein 22% of spermatophyte species are endemic to the region (Lopez-Alvarado and Farris, 2022). In bryophytes, by comparison, there are 23 moss species endemic to the Mediterranean (Hodgetts and Lockhart, 2020) out of 1168 moss species in the area (Ros et al., 2013), resulting in an endemism rate of <2%, and none of the 403 liverwort and hornwort species occurring in the Mediterranean (Ros et al., 2007) is endemic.

In agreement with our fourth hypothesis, the spatial genetic structure of spermatophytes was significantly stronger than those of bryophytes based on the analysis of cpDNA, but not nDNA. Seeds, which carry cpDNA, are in fact several orders of magnitude larger than bryophyte spores. The latter typically measure 10-20 µm on average (Patiño and Vanderpoorten, 2018) and are much smaller than even the smallest seeds, whose size ranges between 0.05 and 6 mm (Arditti and Ghani, 2000). Consequently, the settling velocity, a key parameter of the ability of a particle to disperse in the air (Katul et al., 2005), is much lower in bryophyte spores, wherein it ranges between 0.49 and 8.5 cm/s (Zanatta et al., 2016), than in seeds, wherein, even in ‘dust’ orchid seeds or in seeds with anemochorous adaptations, such as in Asteraceae, settling velocities range between 9 and 40 cm/s (Zotz et al., 2016) and 0.25 and 1.01 m/s (Andersen, 1992). Pollen grains, in contrast, typically measure 20-40 µm, ranging between 10 and about 100 µm (Hao et al., 2020) and exhibit settling velocities of a few cm/s (Hirose and Osada, 2016), i.e., within the range reported for bryophyte spores. The similar genetic structure observed for bryophyte spores and pollen grains, which both carry nDNA, is thus consistent with their similarity in size and dispersal capacities.

## Conclusion and perspectives

The comparative analysis of the genetic structure between northern and southern Europe in bryophytes and spermatophytes presented here suggests that mountain ranges of the Alps and the Pyrenees were a significantly more severe impediment for post-glacial migrations in the latter than in the former. The higher genetic structures found in European spermatophytes as compared to bryophytes offers an appealing explanation for the strikingly lower rates of endemism that characterize bryophytes, especially in areas recognized as biodiversity hotspots such as the Mediterranean. A lower dispersal capacity of spermatophytes has also implications for their ability to migrate as a response to ongoing climate change. The results presented here must, however, be interpreted with caution, as our review revealed a quite limited number of phylogeographic studies for plant species distributed across southern and northern Europe based on DNA sequence variation. In particular, the number of phylogeographic studies for bryophytes in the Mediterranean is extremely limited. Nevertheless, our results reveal intriguing patterns regarding the relative dispersal capacities of pollen grains, spores and seeds, opening the door to a large-scale comparative analysis based on large numbers of markers that can now relatively easily be generated with NGS techniques.

## Data availability statement

Publicly available datasets were analyzed in this study. This data can be found here: https://doi.org/10.1111/j.1095-8339.2007.00775.x; https://doi.org/10.1111/ele.13254; https://doi.org/10.1111/j.1469-8137.2006.01870.x; https://doi.org/10.1111/j.1365-294X.2005.02848.x; https://doi.org/10.1111/mec.13348; https://doi.org/10.1111/j.1469-8137.2010.03328.x; https://doi.org/10.1111/j.1365-294X.2007.03615.x; https://doi.org/10.1002/ece3.3774; https://doi.org/10.1111/j.1365-294X.2009.04449.x ;https://doi.org/10.1111/jbi.13726; https://doi.org/10.1093/botlinnean/boab043; https://doi.org/10.1093/botlinnean/boab043 ;https://doi.org/10.1111/j.1365-294X.2007.03228.x; https://link.springer.com/article/10.1007/s00606-016-1299-1#Sec12; https://doi.org/10.1093/botlinnean/boy025; https://doi.org/10.1093/botlinnean/boy032; https://doi.org/10.1016/j.flora.2019.02.012; https://pbsociety.org.pl/journals/index.php/asbp/article/view/asbp.89313;https://doi.org/10.1371/journal.pone.0179961; https://doi.org/10.1016/j.ppees.2018.10.003.

## Author contributions

TF: Writing – original draft, Writing – review & editing. AL: Writing – review & editing. FC: Writing – review & editing. AV: Writing – original draft, Writing – review & editing, Conceptualization.

## Supplementary material

Supplementary Table 1 Datasets employed in the comparative analysis of the spatial genetic structure of bryophytes and spermatophytes in western Europe: species selected, literature source, number of loci and sample size (southern-northern).

Supplementary Table 2 Slope, p-value, and mean ± 1.96 SD of the slope after Jackknife across loci (for multilocus data, NA otherwise) of the regression between pairwise kinship coefficient Fij derived from cpDNA sequence data and geographic distances in selected bryophyte and spermatophyte species in northern and southern Europe.
