# Supplementary material for: Dispersal capacities of pollen, seeds and spores: insights from comparative analyses of spatial genetic structures in bryophytes and spermatophytes
Source: Front Plant Sci. 2023 Oct 30;14:1289240. doi: 10.3389/fpls.2023.1289240 (PMC10642818; doi:10.3389/fpls.2023.1289240)

Supplementary Material

**Supplementary Figures S1-S28.** Distribution of cpDNA and nDNA alleles in 11 bryophyte and 17 spermatophyte species across western Europe.

S1. Antitrichia curtipendula


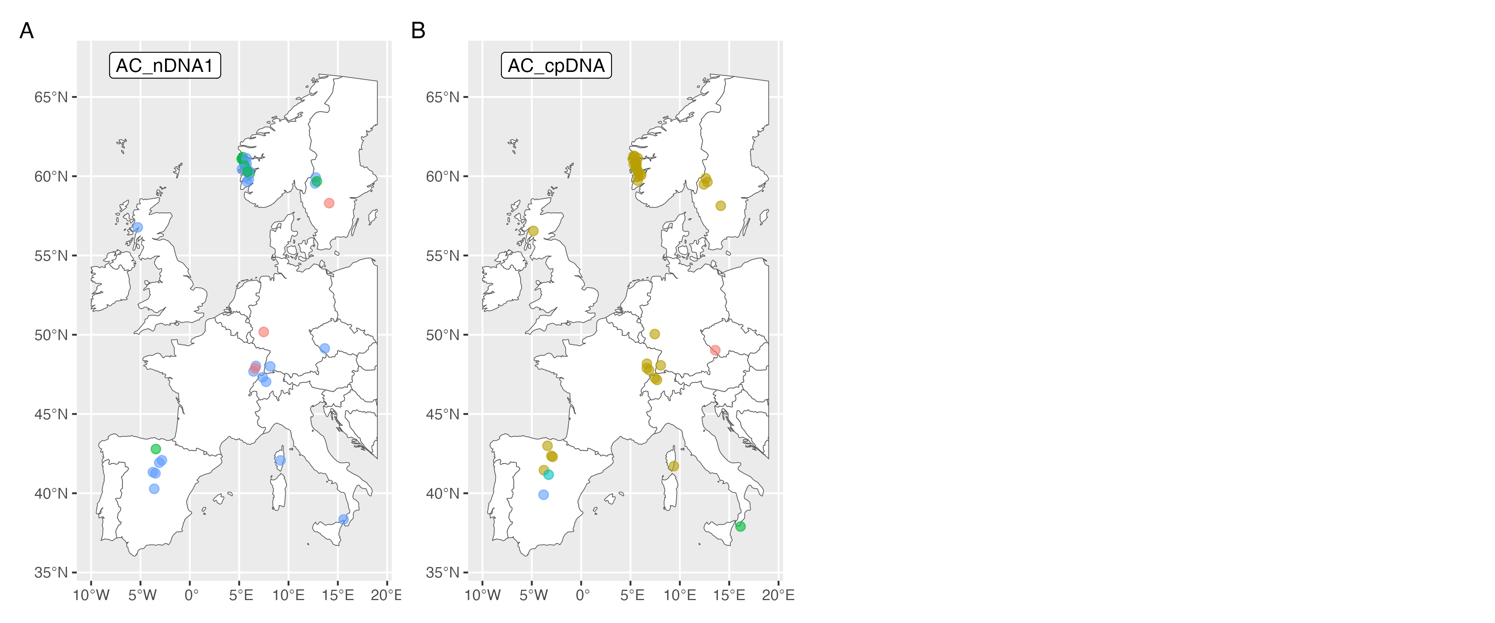


S2. Amphidium mougeotii


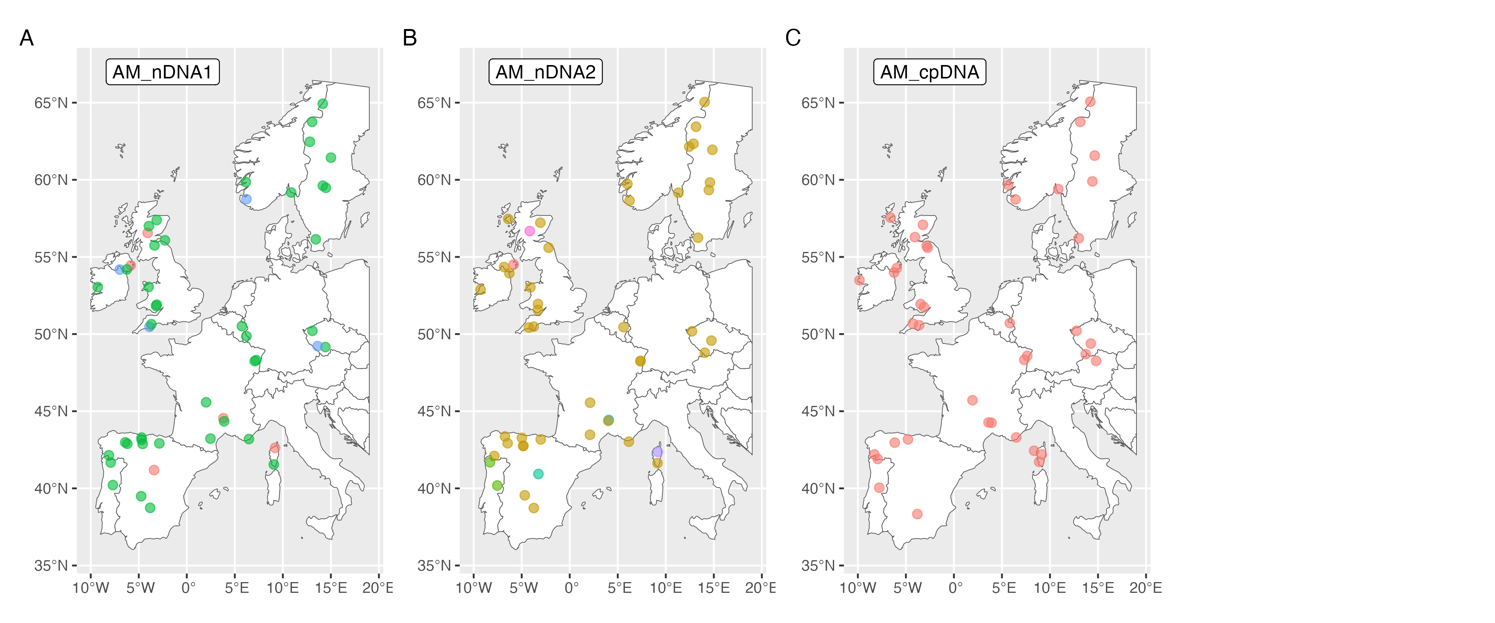


S3. Calypogeia fissa


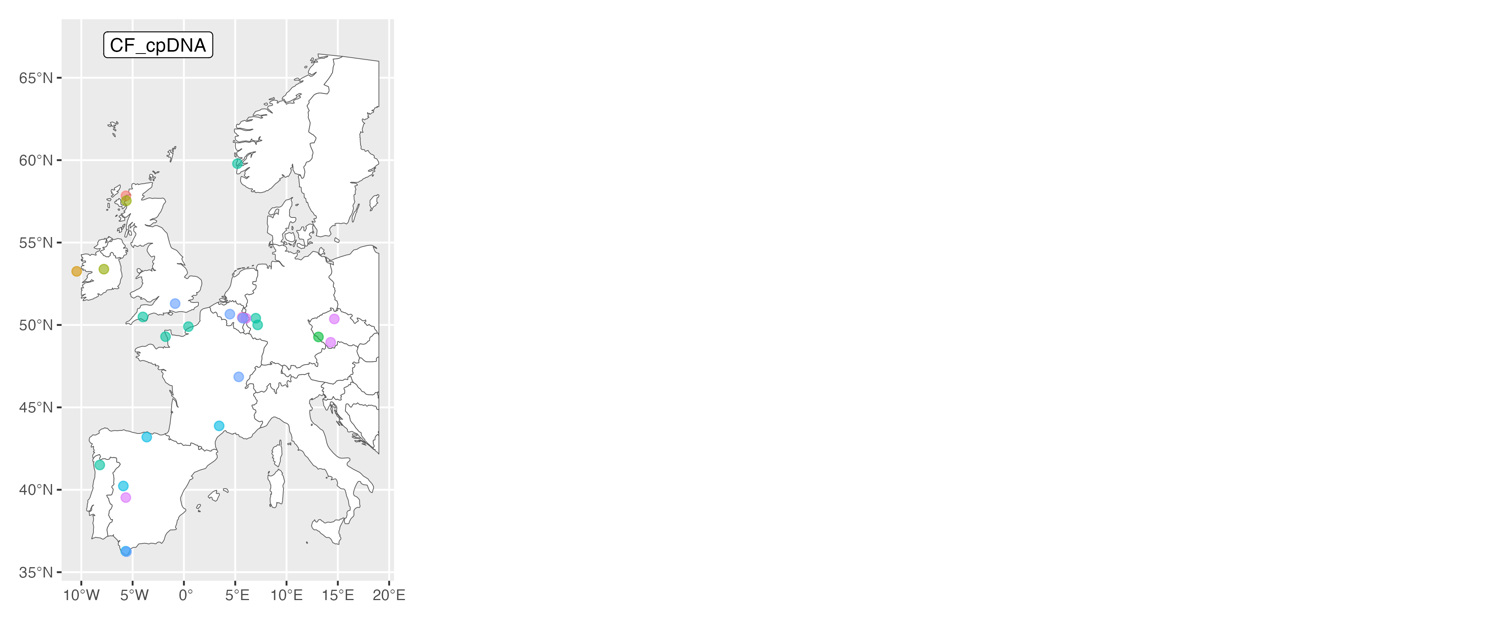


S4. Homalothecium sericeum
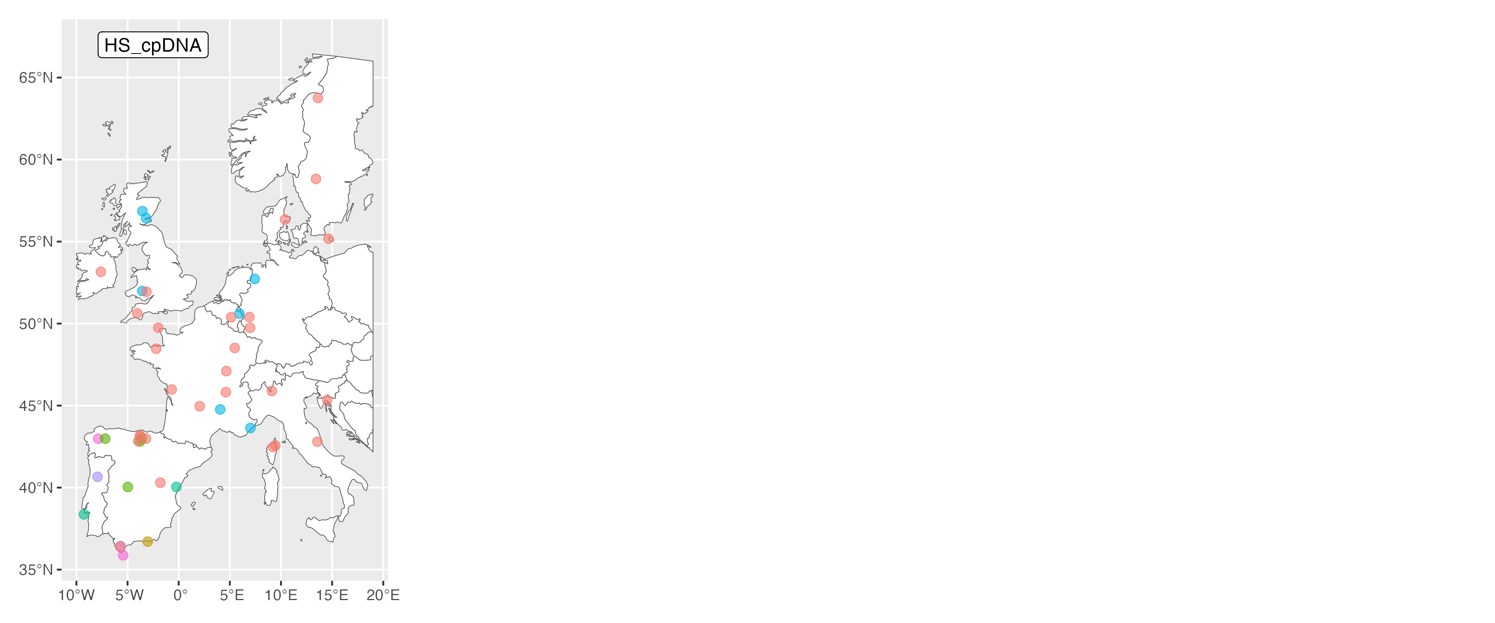


S5. Metzgeria furcata


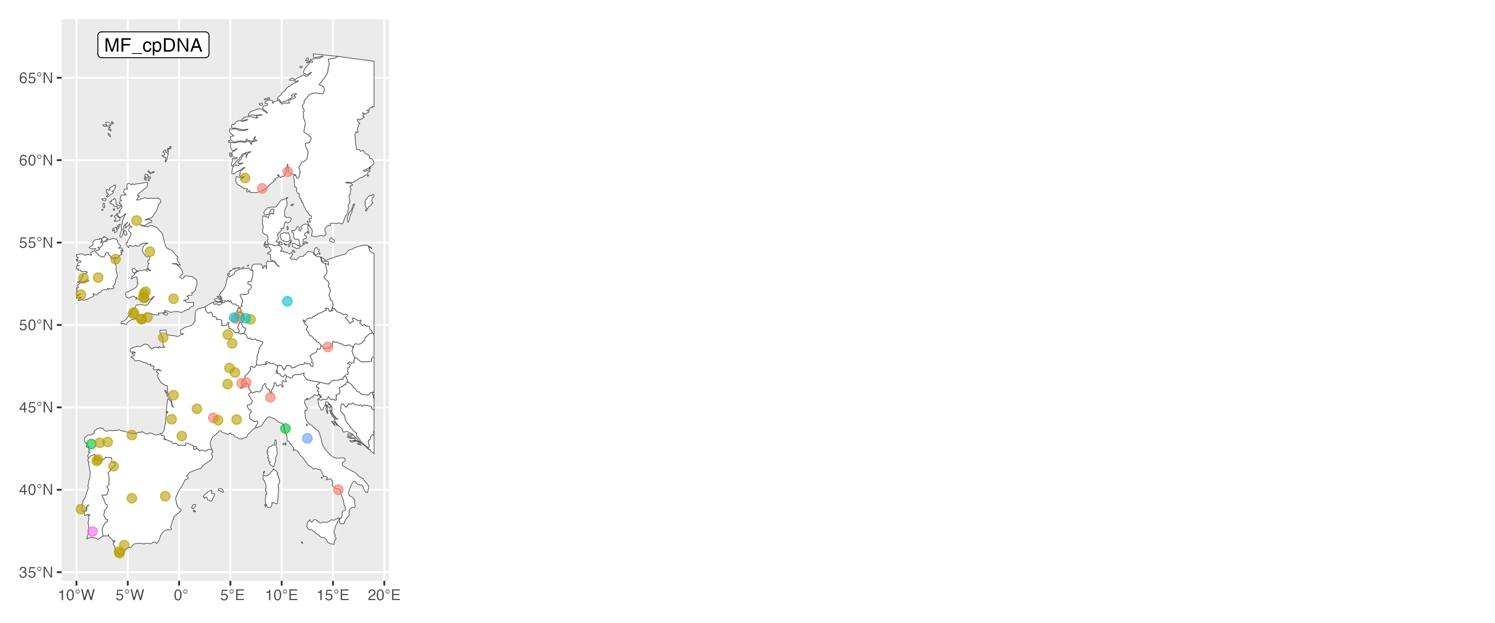


S6. Lewinskya affine (previously Orthotrichum affine)
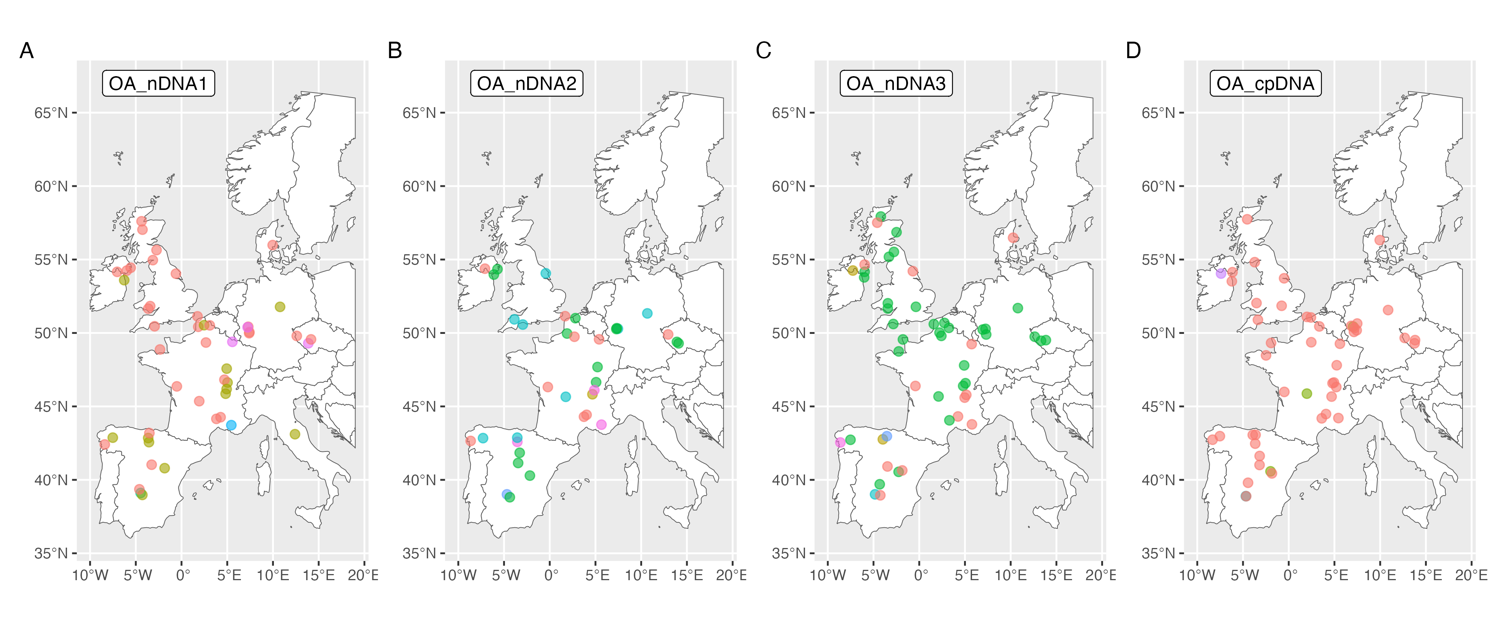


S7. Pulvigera lyellii (previously Orthotrichum lyellii)


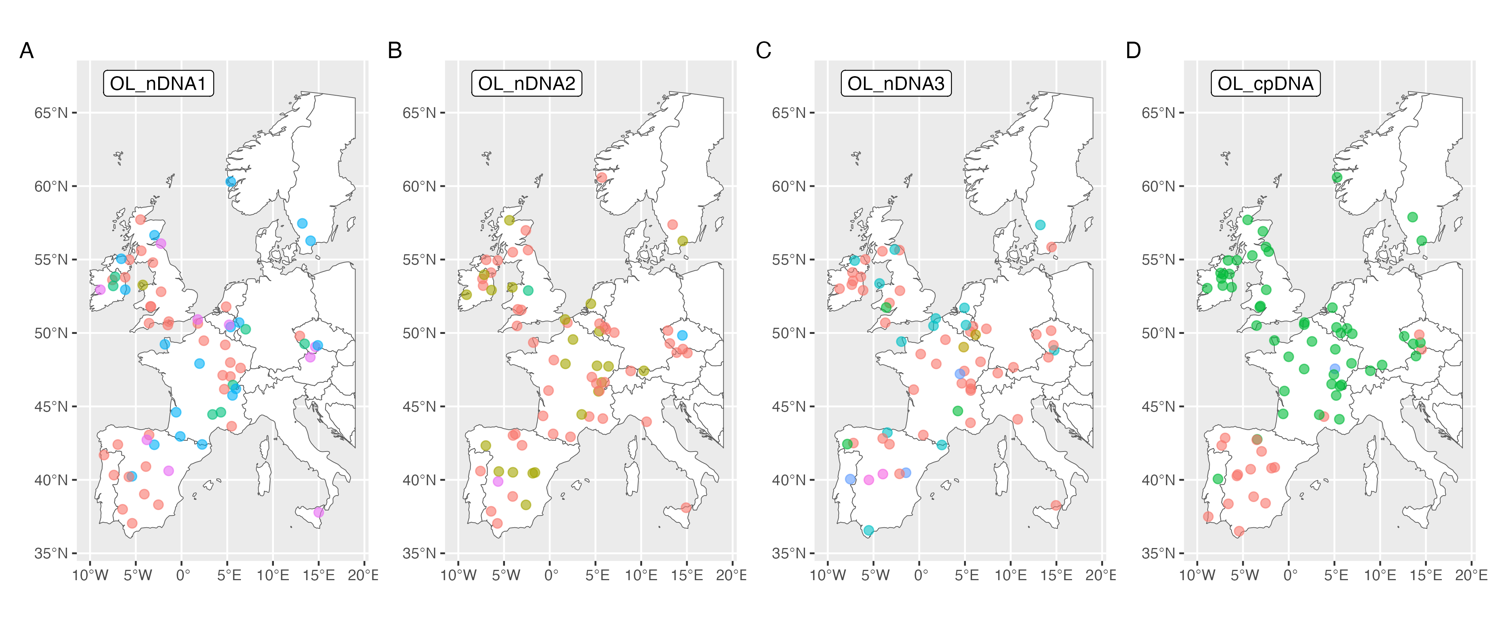


S8. Plagiomnium undulatum


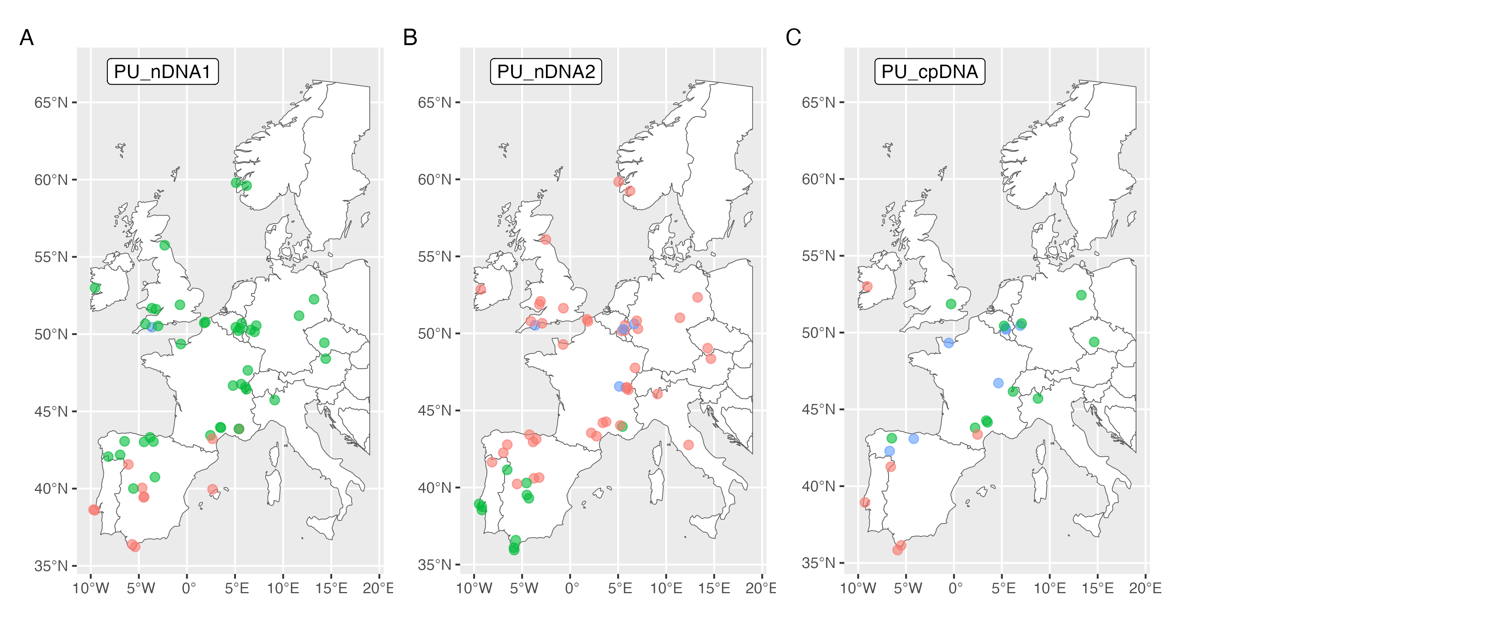


S9. Plagiothecium undulatum


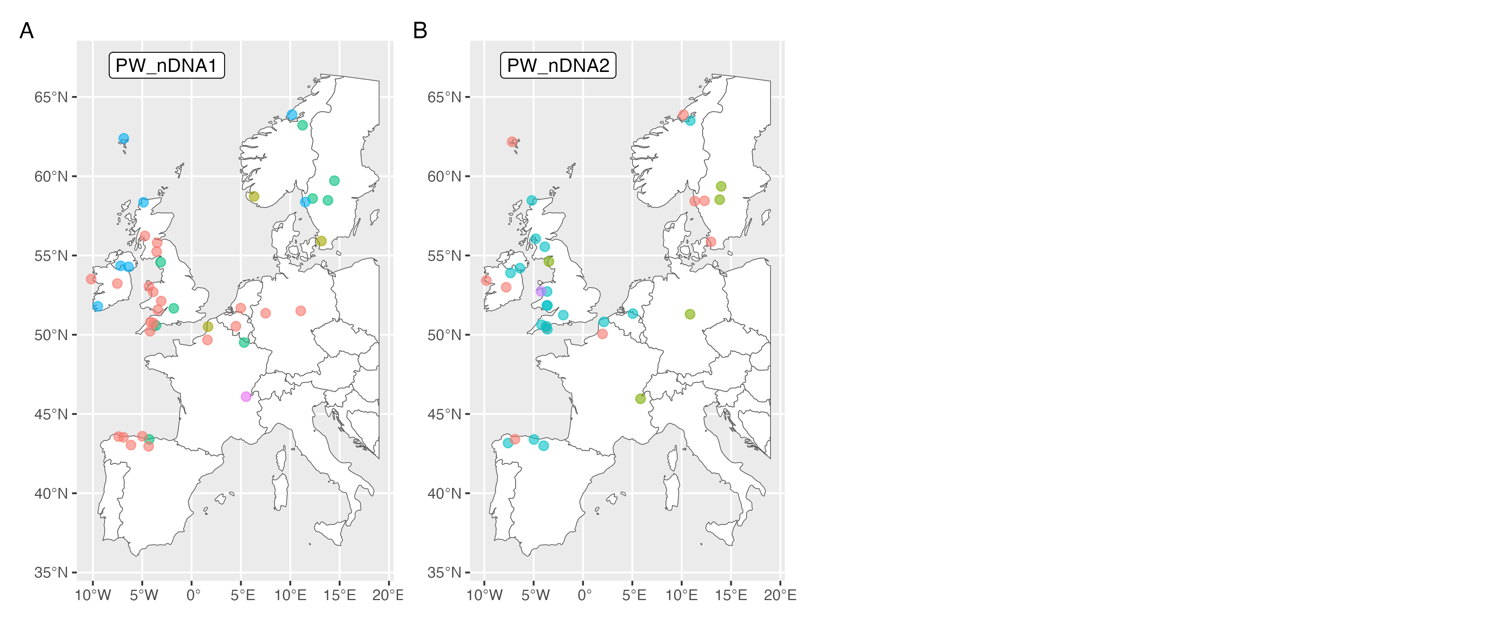


S10. Scorpiurium circinatum
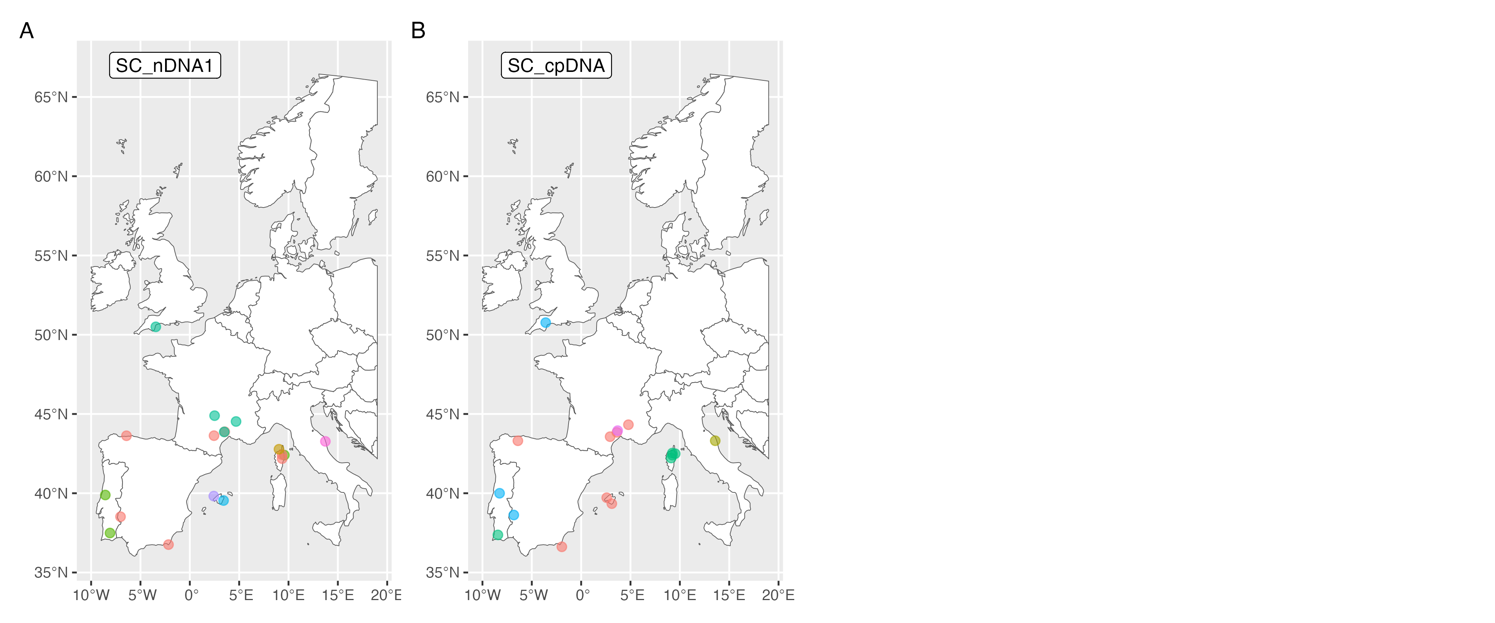


S11. Sphagnum fimbriatum


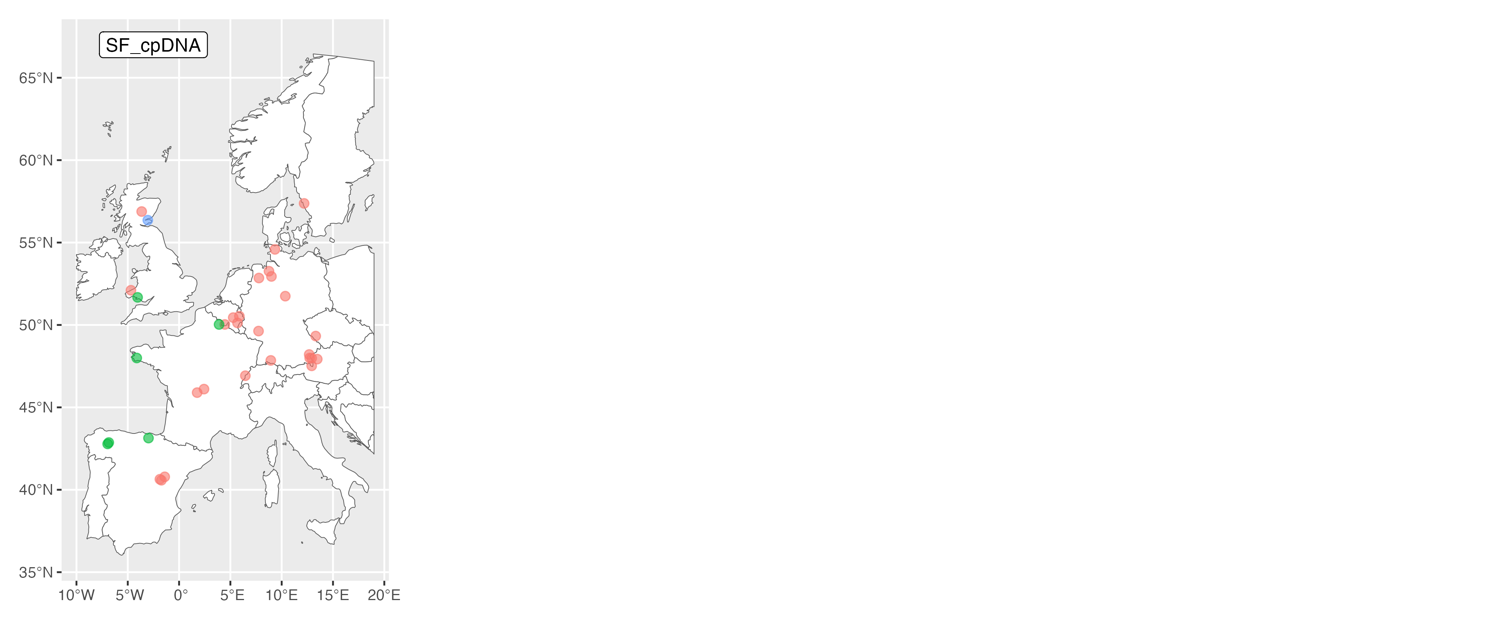


S12. Arabis alpina


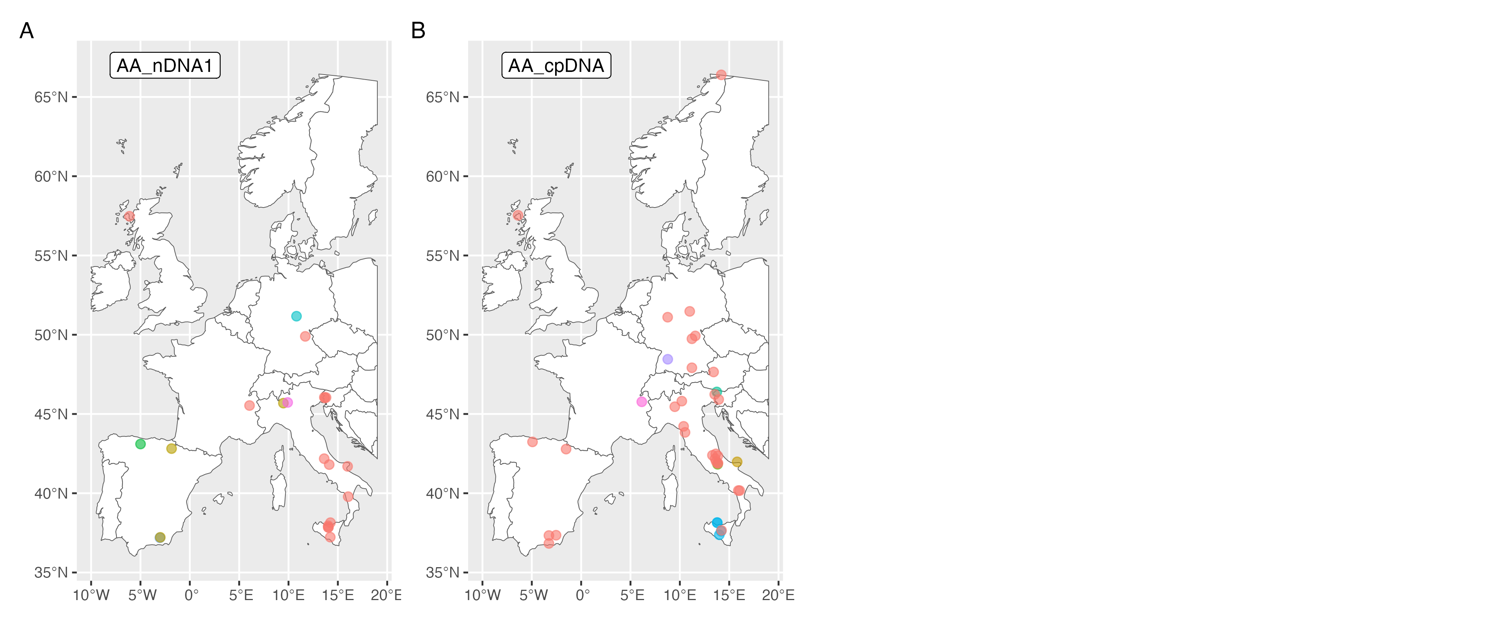


S13. Alnus glutinosa


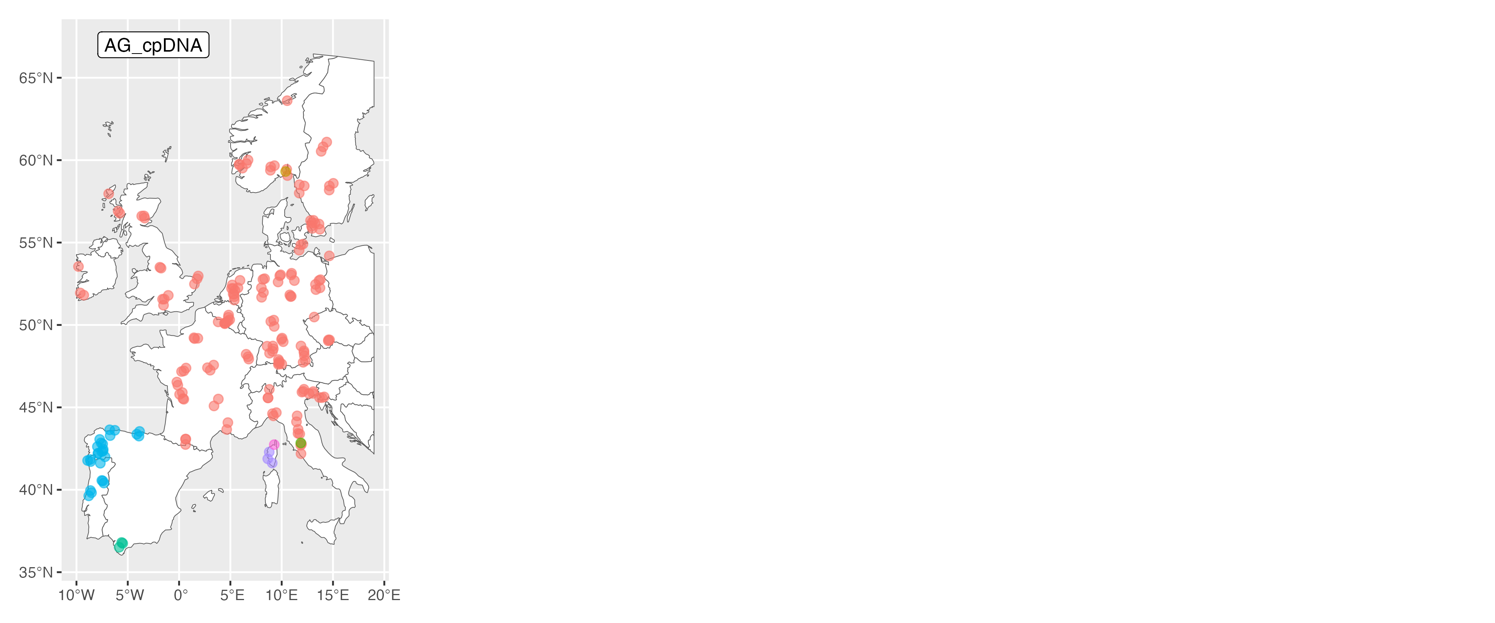


S14. Aegilops geniculata
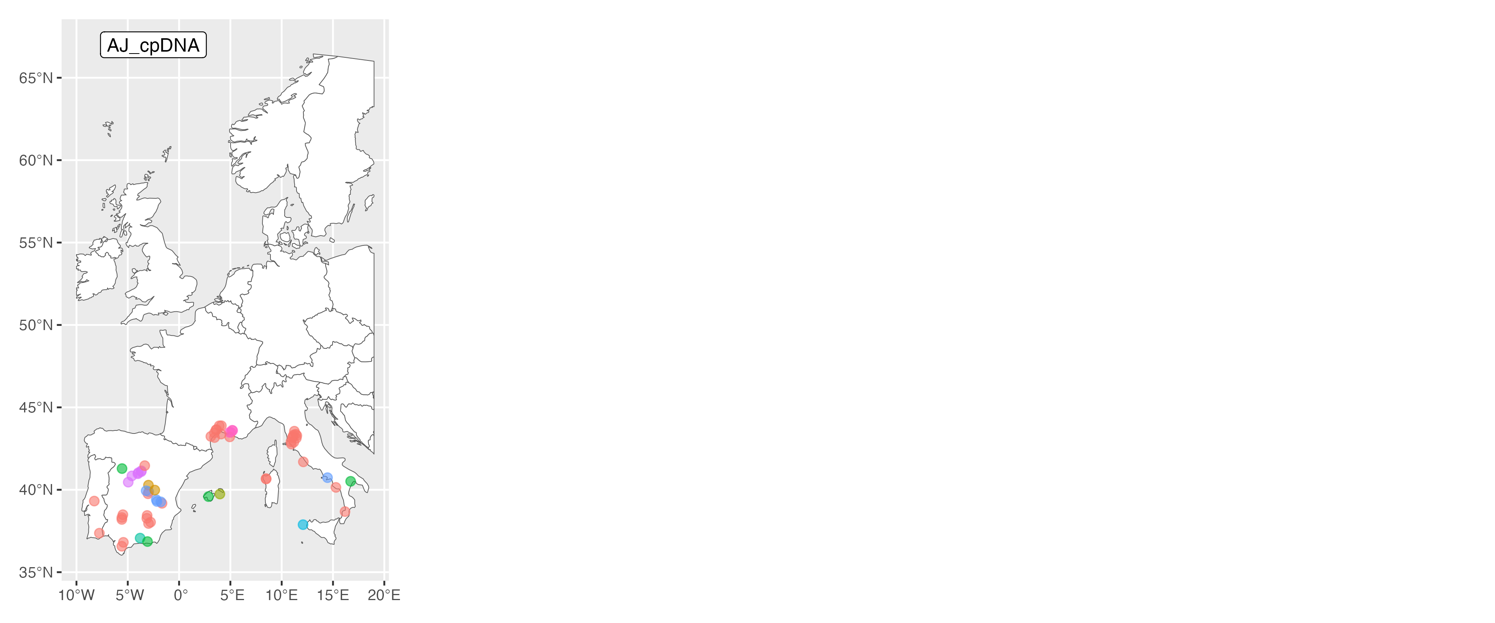


S15. Arabidopsis thaliana


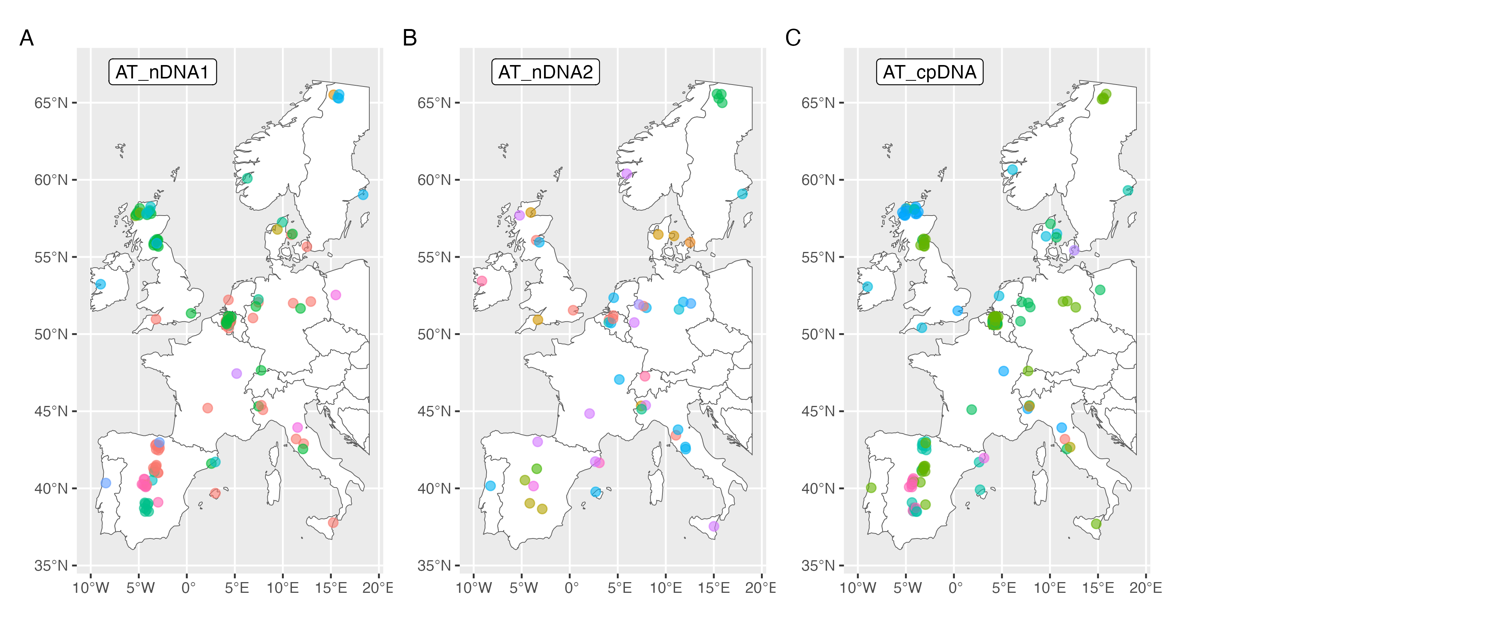


S16. Beta vulgaris maritima


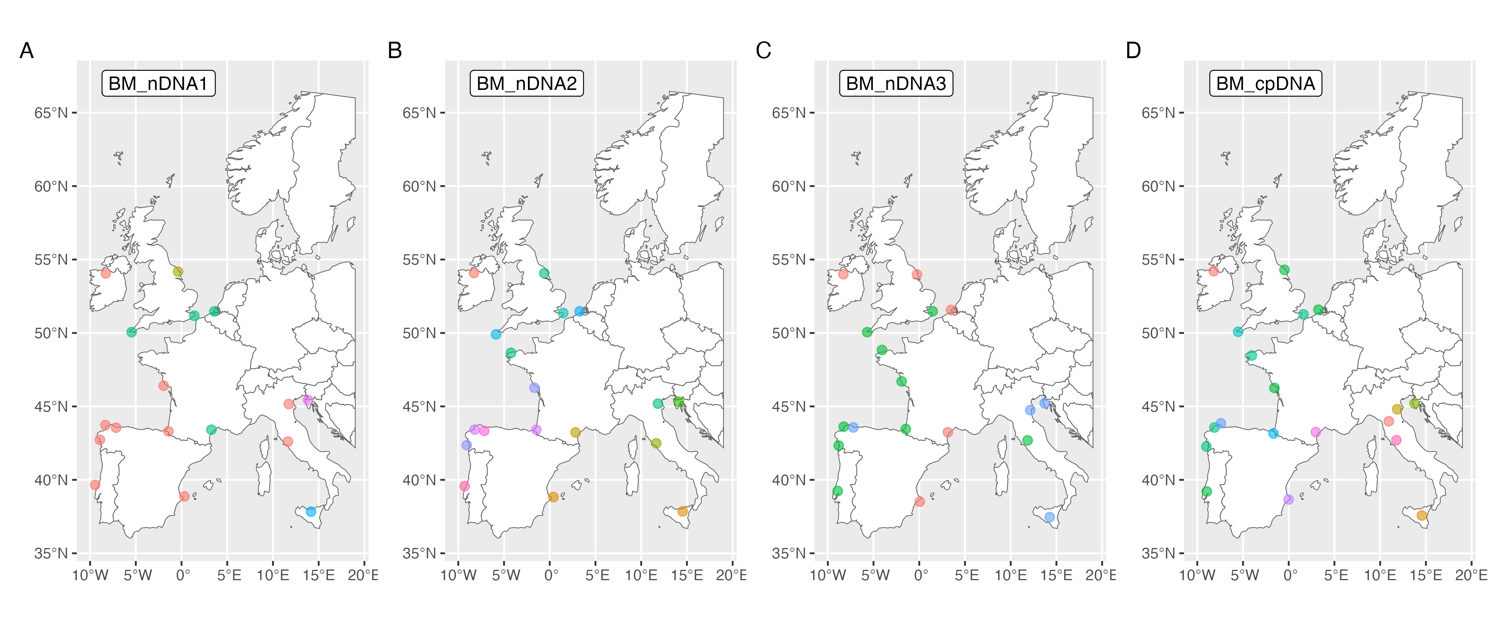


S17. Carex extensa


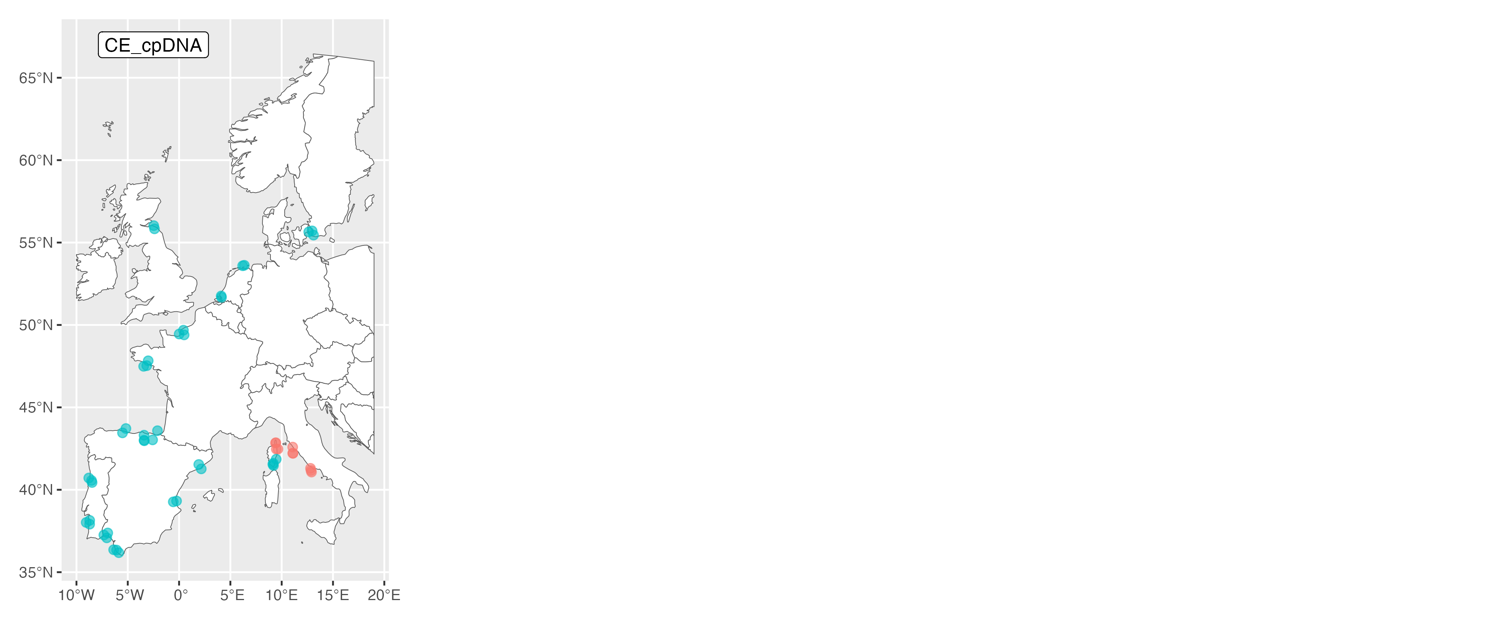


S18. Ceratonia siliqua


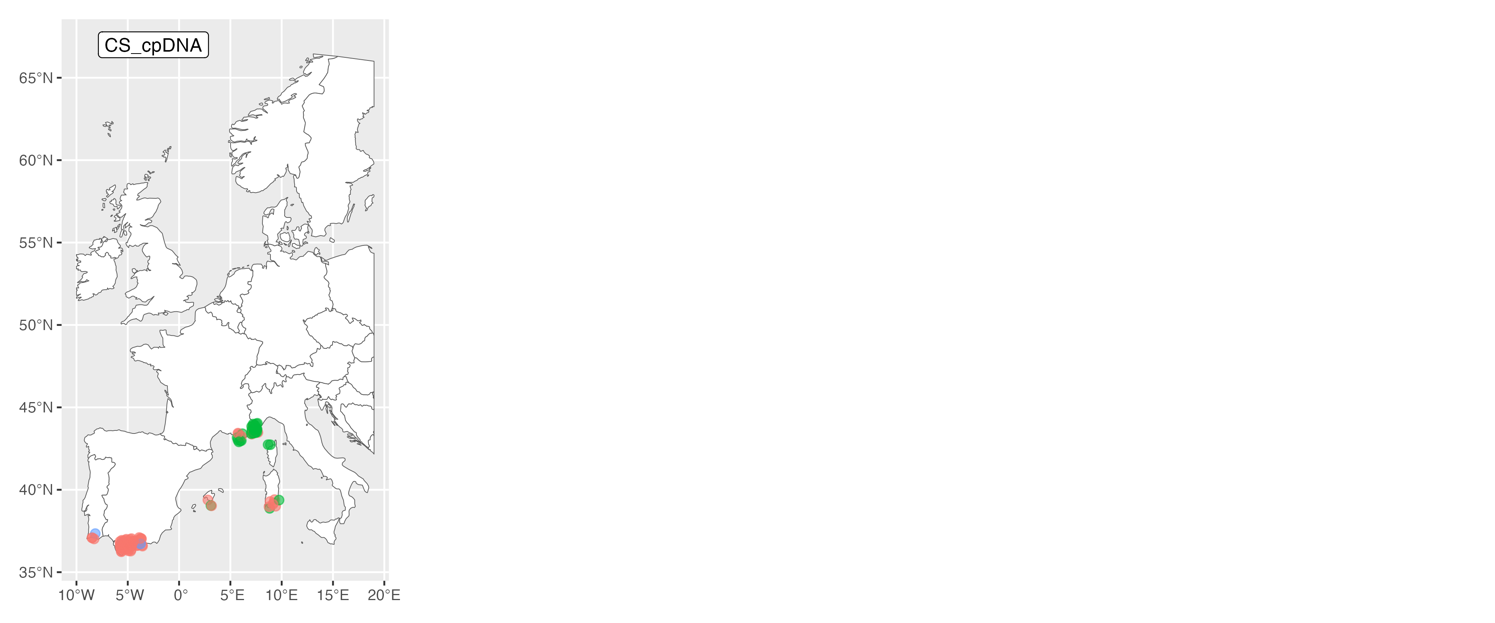


S19. Calluna vulgaris


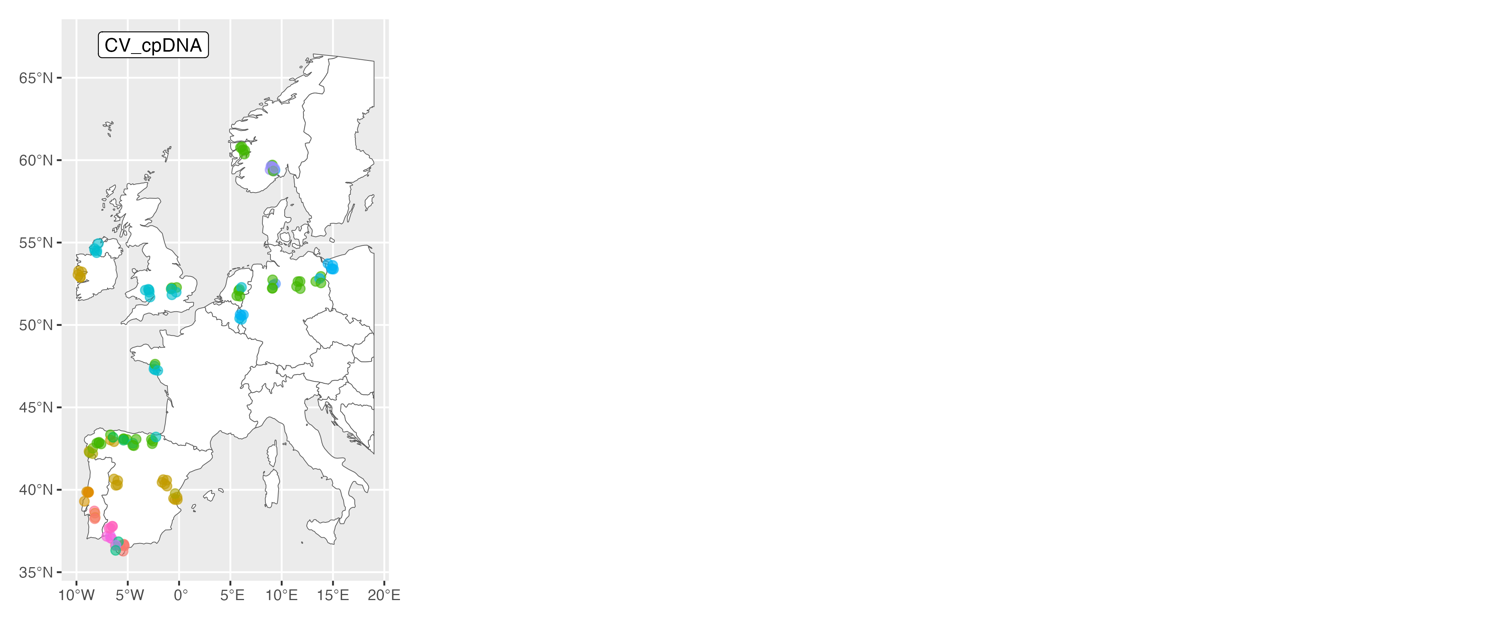


S20. Hedera helix


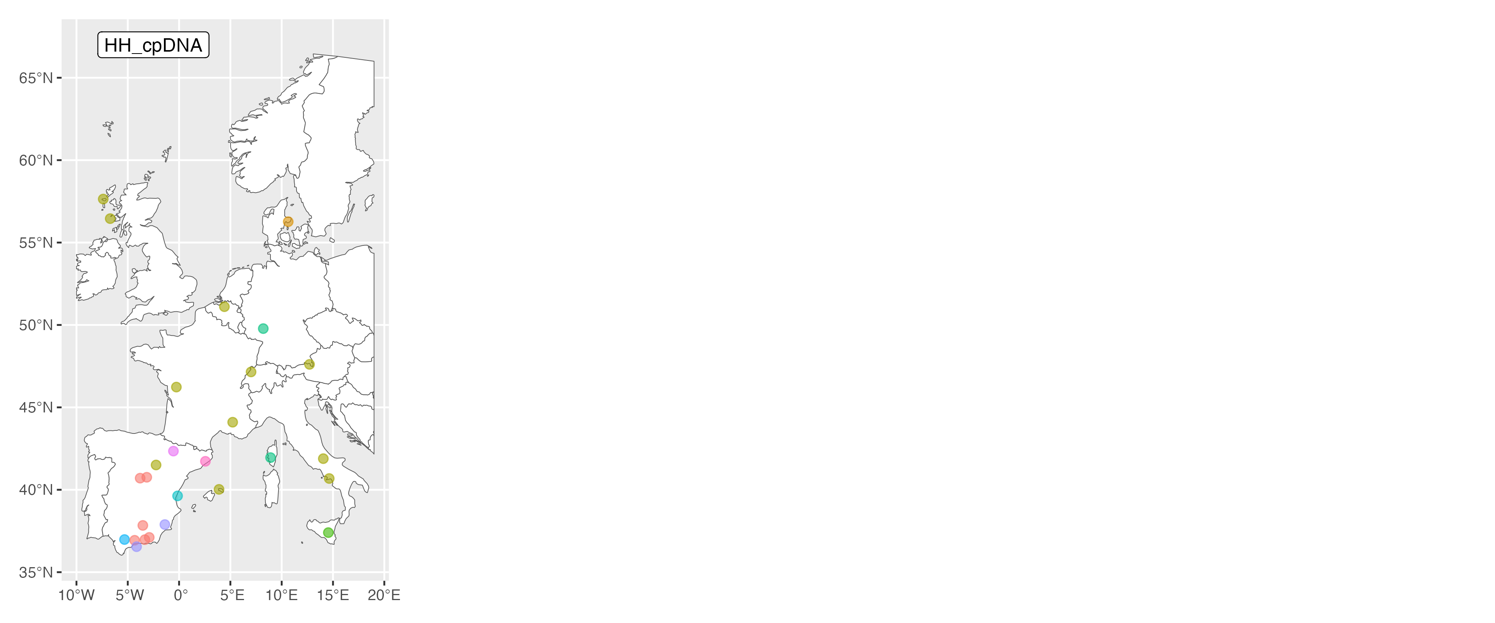


S21. Hordeum marinum


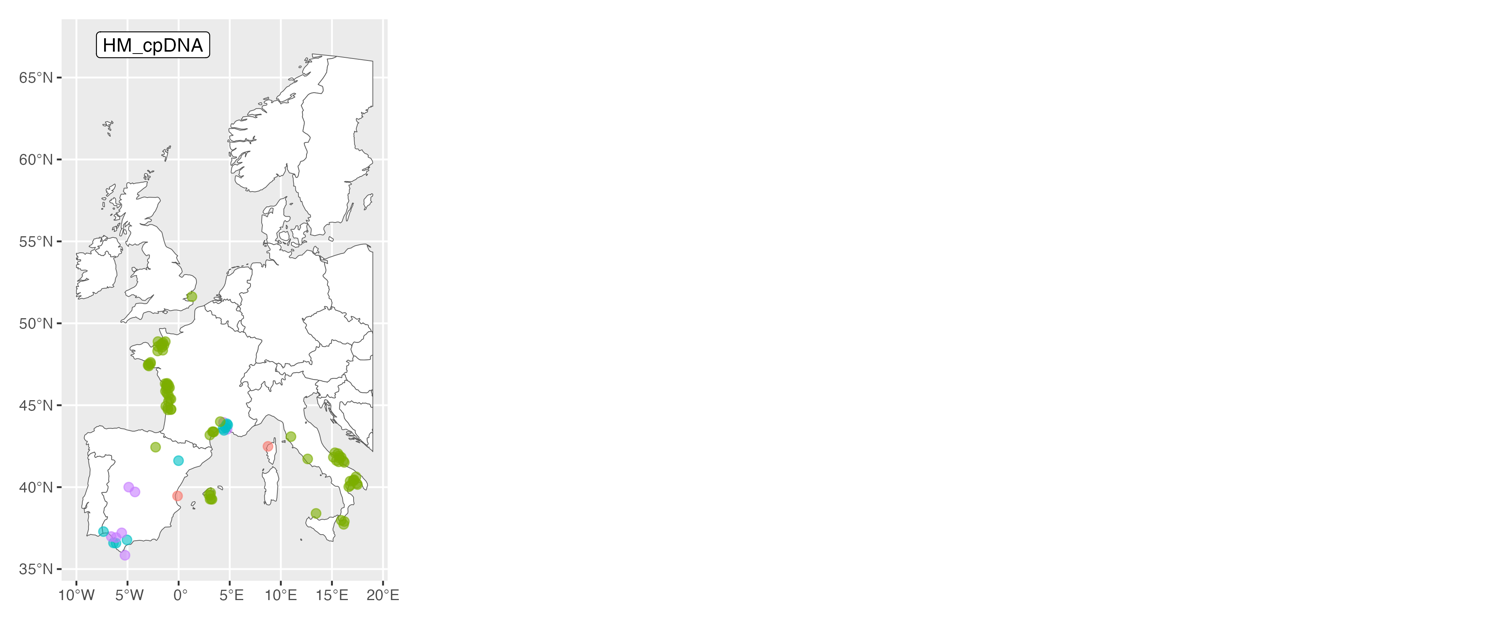


S22. Helianthemum nummularium


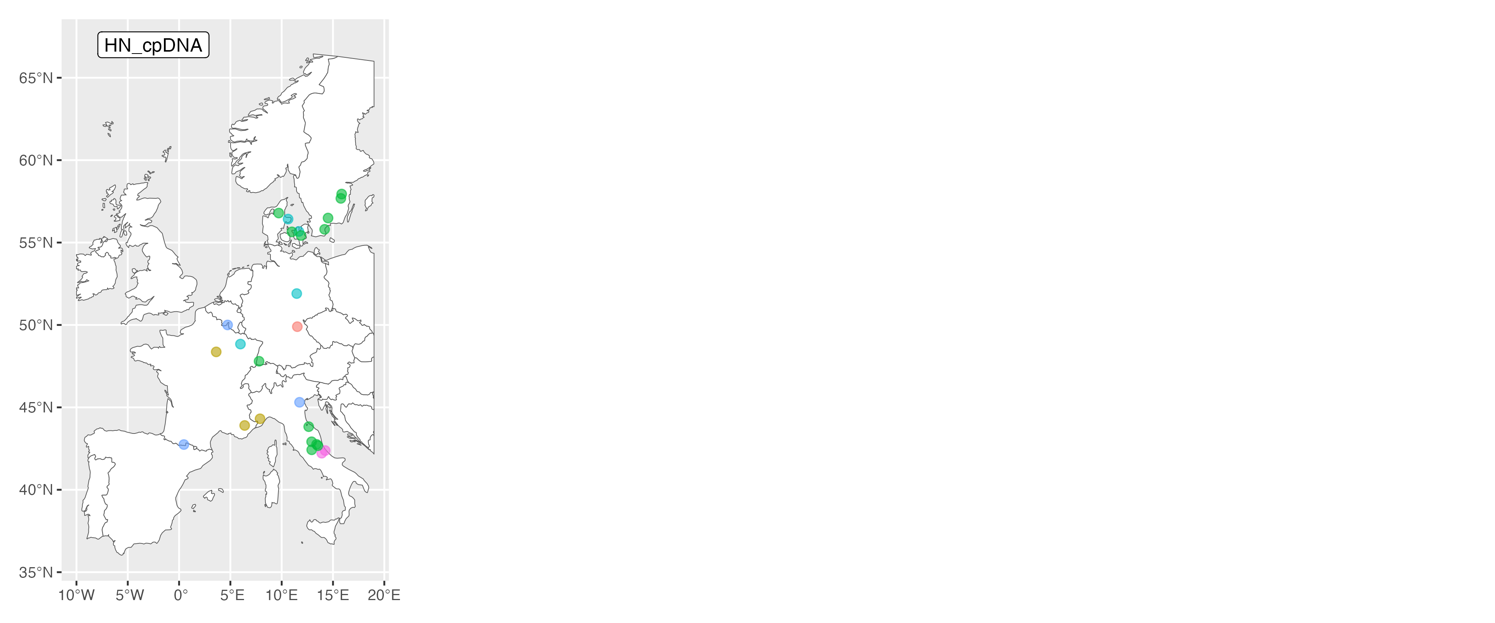


S23. Lavatera maritima


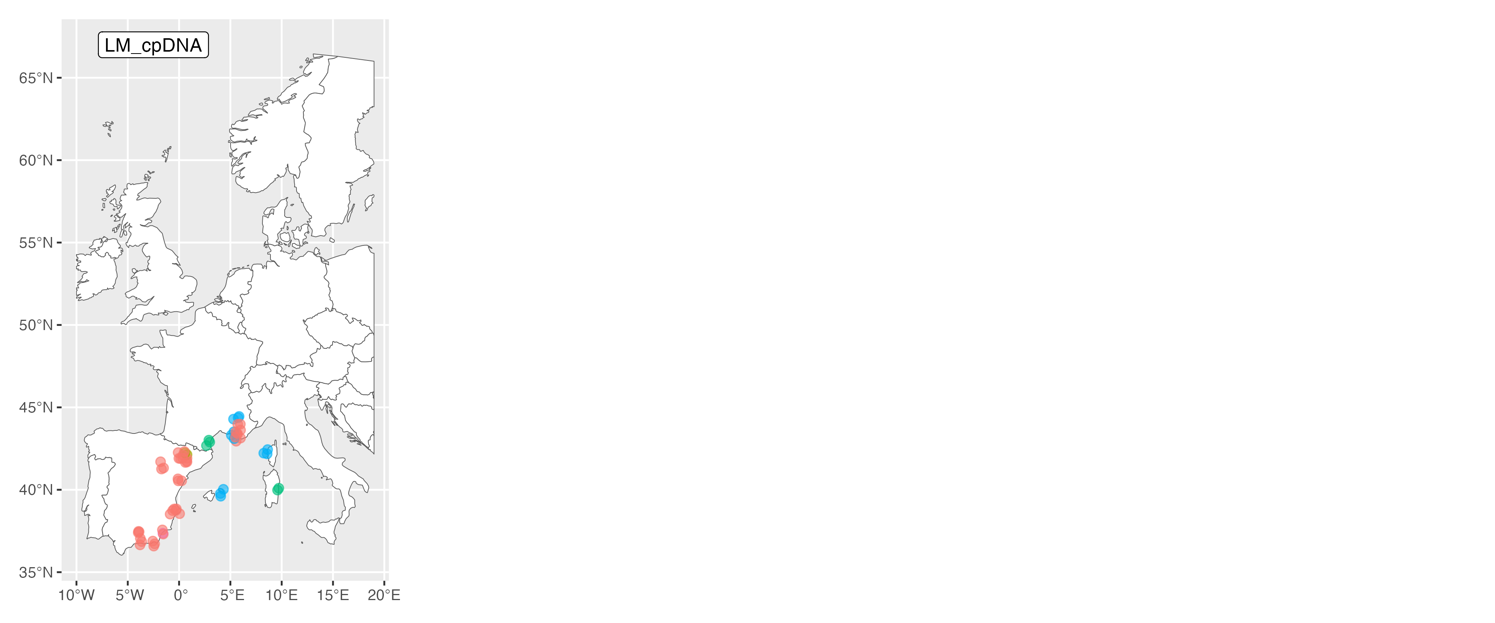


S24. Myrtus communis


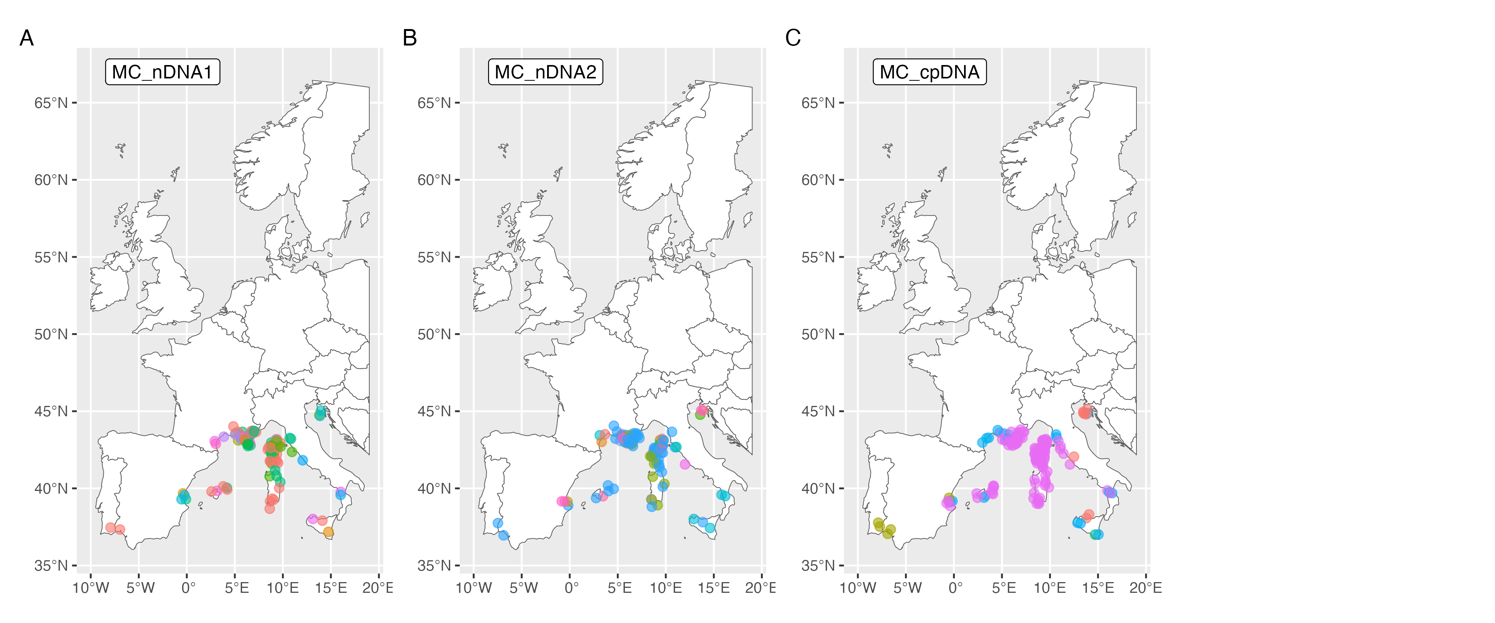


S25. Microthlaspi perfoliatum


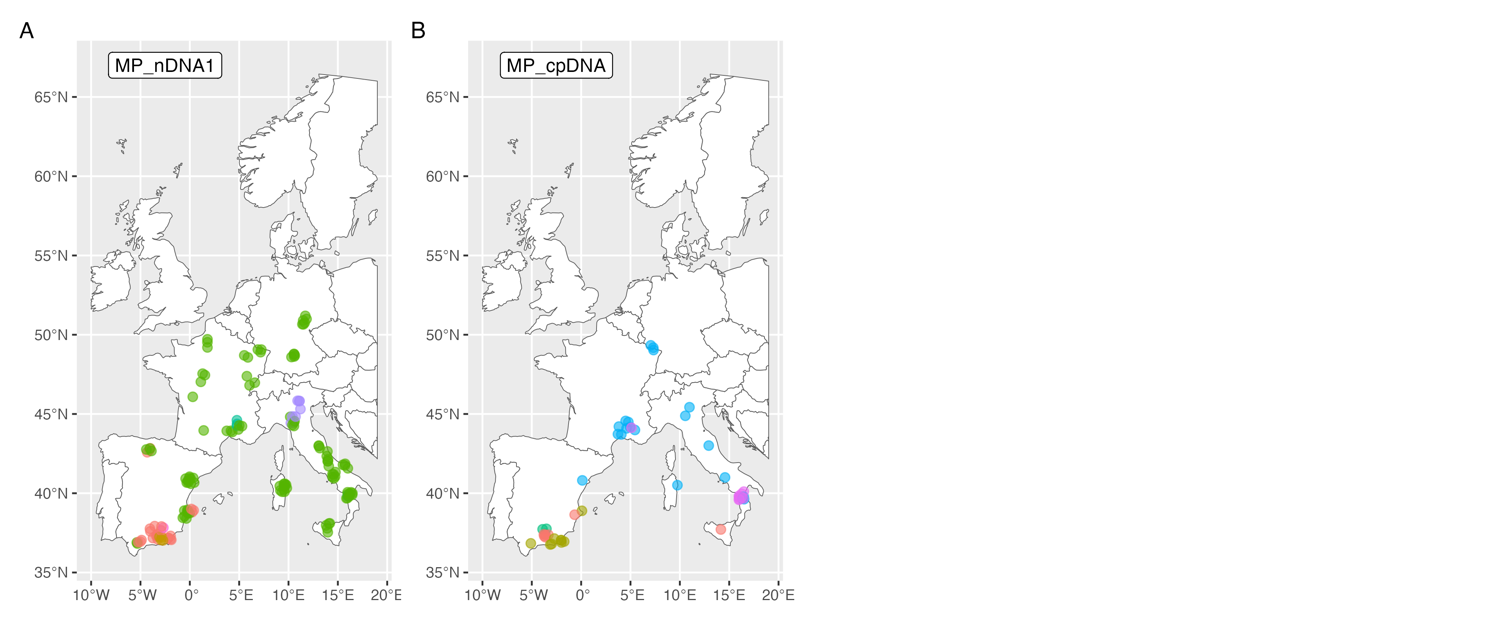


S26. Primula vulgaris


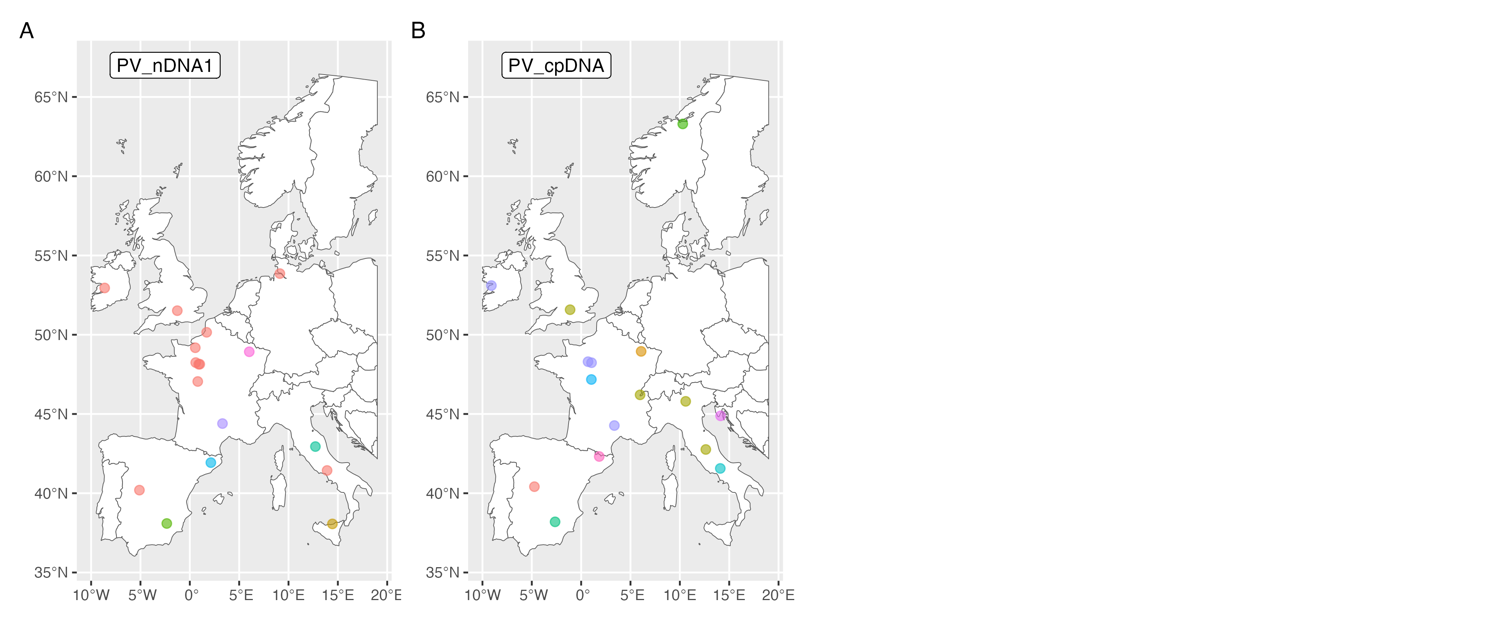


S27. Sedum album


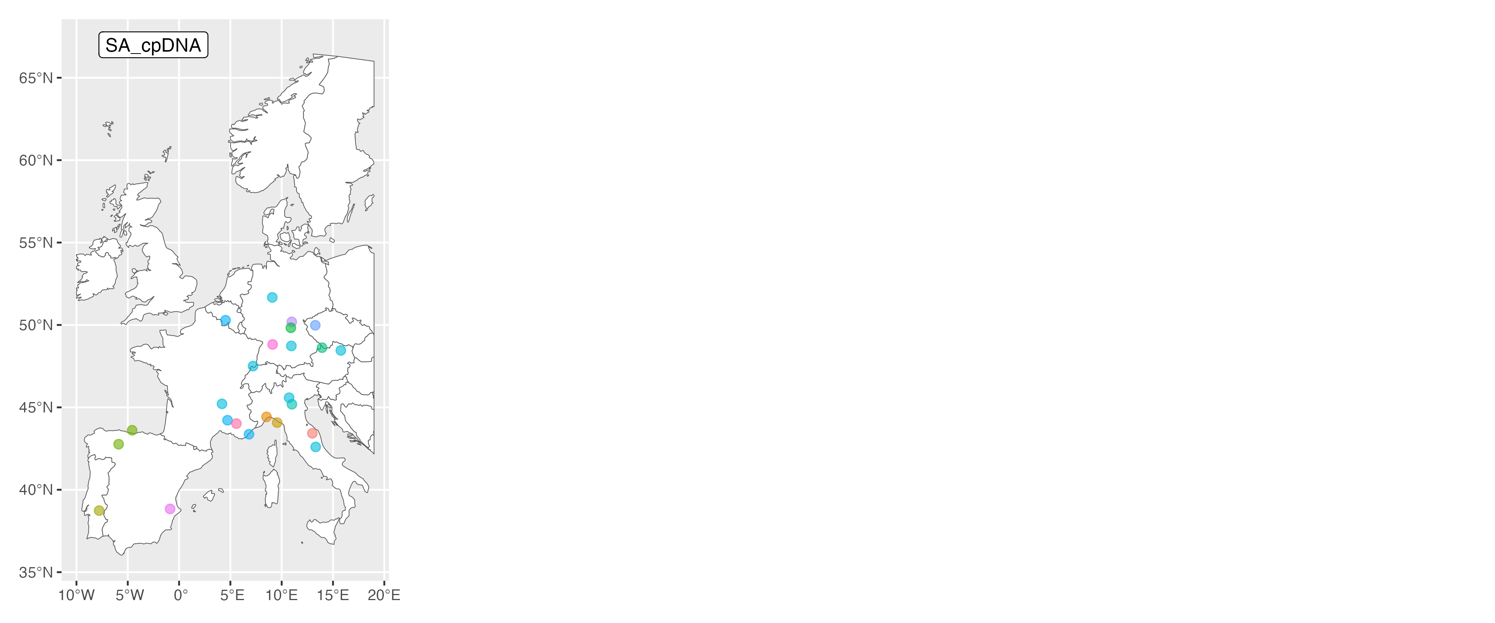


S28. Silene nutans


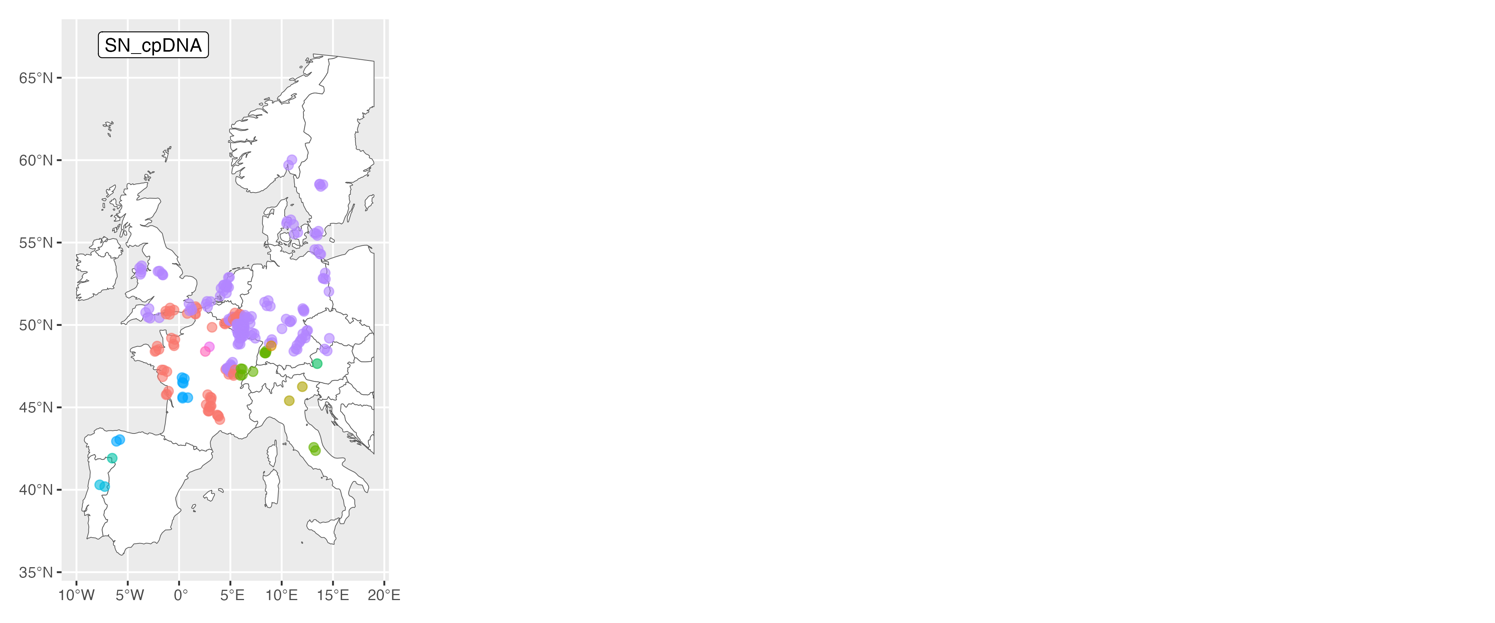

Supplement: Supplementary file 3 [file DataSheet_1.docx]
